# Upregulation of *CISD2* augments ROS homeostasis and contributes to tumorigenesis and poor prognosis of lung adenocarcinoma

**DOI:** 10.1038/s41598-017-12131-x

**Published:** 2017-09-19

**Authors:** Shih-Miao Li, Chung-Hsing Chen, Ya-Wen Chen, Yi-Chen Yen, Wen-Tsen Fang, Fang-Yu Tsai, Junn-Liang Chang, Ying-Ying Shen, Shiu-Feng Huang, Chih-Pin Chuu, I-Shou Chang, Chao A. Hsiung, Shih Sheng Jiang

**Affiliations:** 1 Institute of Bioinformatics and Structural Biology, National Tsing Hua University, Hsinchu, Taiwan; 2 Institute of Population Health Sciences, National Health Research Institutes, Miaoli, Taiwan; 3 National Institute of Cancer Research, National Health Research Institutes, Miaoli, Taiwan; 4 Department of Pathology & Laboratory Medicine, Taoyuan Armed Forces General Hospital, Taoyuan, Taiwan; 5 Biomedical Engineering Department, Ming Chuang University, Taipei, Taiwan; 6 Pathology Core Laboratory, National Health Research Institutes, Miaoli, Taiwan; 7 Institute of Molecular and Genomic Medicine, National Health Research Institutes, Miaoli, Taiwan; 8 Institute of Cellular and System Medicine, National Health Research Institutes, Miaoli County, Taiwan; 9 Graduate Program for Aging, China Medical University, Taichung City, Taiwan

## Abstract

*CISD2* is a redox-sensitive gene critical for normal development and mitochondrial integrity. *CISD2* was known to have aberrant expression in several types of human cancers. However, its relation with lung cancer is still not clear. In this study we found *CISD2* mRNA was significantly upregulated in lung adenocarcinoma (ADC) samples, compared with their adjacent normal counterparts, and was correlated with tumor stage, grade, and prognosis based on analysis of clinical specimens-derived expression data in public domain and our validation assay. Cell based assay indicated that *CISD2* expression regulated accumulation of reactive oxygen species (ROS), polarization of mitochondrial membrane potential, as well as cell viability, apoptosis, invasiveness, and tumorigenicity. In addition, *CISD2* expression was found significantly correlated with stress response/redox signaling genes such as *EGR1* and *GPX3*, while such correlations were also found valid in many public domain data. Taken together, upregulation of CISD2 is involved in an increased antioxidant capacity in response to elevated ROS levels during the formation and progression of lung ADC. The molecular mechanism underlying how CISD2 regulates ROS homeostasis and augments malignancy of lung cancer warrants further investigations.

## Introduction

Lung cancers are a major cause of cancer-related deaths globally, mostly attributable to late diagnosis and lack of efficient treatment^1^. Given such a high mortality, it is critical that we improve our understanding of how lung cancers arise and progress so that cancer prevention strategies can be implemented or biomarkers and therapeutic targets can be developed to improve early detection and treatment.

The most well-known risk factor for lung cancer is cigarette smoking^2,3^. Many components of cigarette smoke induce oxidative stress by transmitting or generating reactive oxygen species (ROS)^4^. ROS are also produced endogenously in cells, where mitochondria have been conventionally recognized as one of the sources of intracellular ROS^5^. Additionally, ROS are generated in response to external stimuli, such as inflammatory cytokines, chemotherapeutic drugs, and ionizing radiation. Having been shown to be genotoxic, ROS also functions as signaling molecules^6^. ROS are known to play a pivotal role in carcinogenesis by promoting proliferation, invasiveness, and metastasis, and by inhibiting apoptosis. Alternatively, they can also play anticarcinogenic roles, e.g., by inducing cell cycle arrest, apoptosis, and necrosis^7,8^. As with all other types of human cancer, the role of ROS/oxidative stress in lung cancer seems important but complicated.

In an initiative to scrutinize the roles of oxidative stress-related genes during tumorigenesis or progression of lung cancer, we noticed the upregulation of *CISD2* (CDGSH iron sulfur domain 2) in lung adenocarcinoma (ADC). CISD2, also known as NAF-1, ZCD2 or Miner1, belongs to the CDGSH iron sulfur domain protein family. The *CISD2* gene is located on chromosome 4q; it encodes a protein contains a CDGSH domain, a transmembrane domain, and a conserved amino acid sequence for iron binding^9,10^. CISD2 is an essential protein in development. Its dysfunction in humans is known to cause Wolfram syndrome type 2 and *CISD2* knockout mice show a phenotype of premature aging^11^. CISD2 is localized mainly in mitochondria and partially in the endoplasmic reticulum (ER), and has been functionally linked to maintaining mitochondrial integrity and autophagy^12,13^. Although CISD2 activity is critically required for normal development, overexpression of *CISD2* has been linked to several human cancers, including breast cancer^13,14^, cervical cancer^15^, gastric cancer^16^, and laryngeal squamous carcinoma (SQC)^17^, indicating it plays an oncogenic role. Despite these findings, it is still unclear whether CISD2 is associated with lung cancer.

In the present study, we aimed to understand whether *CISD2* expression is associated with the formation of lung ADC, and with the prognosis for patients with this cancer type. We provide several lines of evidence to show that overexpression of *CISD2* is oncogenic to lung cancer. The antioxidant but oncogenic roles of CISD2 in lung cancer are discussed.

## Results

### CISD2 expression is upregulated in lung ADC and linked to poor prognosis

**Figure 1:**
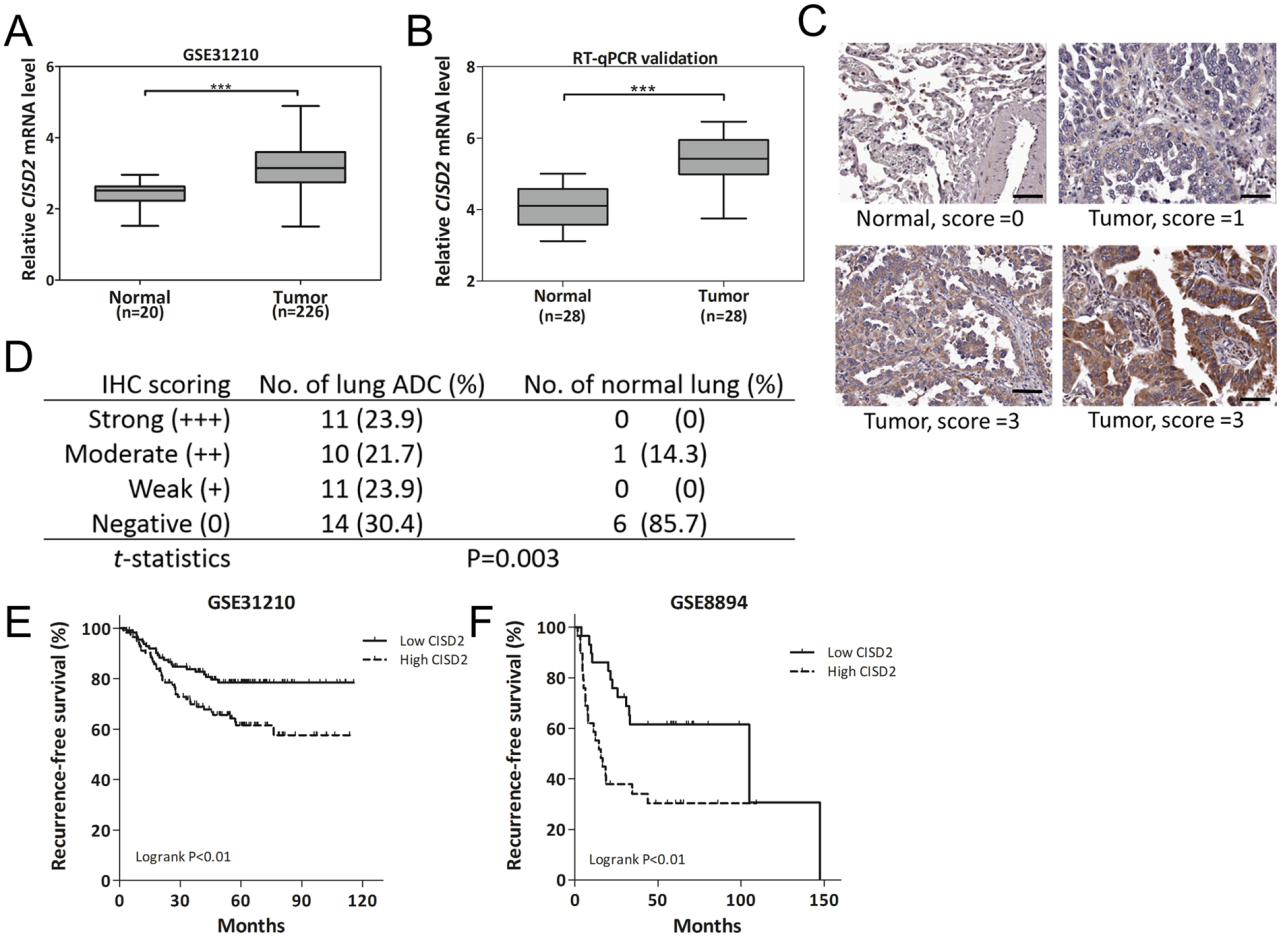
*CISD2* expression is upregulated in lung ADC and associated with poor prognosis. (**A**) Box plot showing upregulation of *CISD2* mRNA expression levels in lung ADC tumor tissues compared with those in normal lung tissues, based on public domain data GSE31210. Data were downloaded from Oncomine and analyzed directly without further processing. (**B**) RT-qPCR assay of *CISD2* mRNA expression of samples from our previously used cohort of 56 specimens containing 28 lung ADC and 28 normal lung tissues (not matched pair). (**C**) Representative images of IHC staining of CISD2 protein performed on normal and lung ADC tissues. Scale bar: 50 μm. (**D**) A summary of IHC analysis of CISD2 protein expression in lung ADC and normal lung tissues in commercial tissue microarrays (c.f. Materials and Methods). (**E** and **F**) Survival analysis on *CISD2* mRNA expression and recurrence-free survival using public domain data. In each dataset, patients were stratified into low CISD2 (solid line) and high CISD2 (dashed line) groups using median *CISD2* expression level as cutoff; significance of difference in survival between groups was estimated using log rank test. For (**A**), (**B**) and (**D**) p values were obtained using Student’s *t*-test, ***P < 0.001. Detailed information about public domain datasets is available in Supplementary Table S1.

To determine whether *CISD2* is aberrantly expressed in human lung ADC and if its expression has any clinical relevance, we first analyzed *CISD2* expression profiles in public lung ADC datasets. We analyzed three independent datasets (GSE31210^18^, GSE27262^19^, and GSE19188^20^), which comprise a total of 406 cases, and found repeatedly elevated *CISD2* mRNA expression in lung ADC tissue samples compared with their adjacent normal counterparts (Figs 1A and S1A). This upregulation of *CISD2* mRNA was also found in our in-house-generated dataset GSE46539 (Supplementary Fig. S1B), and can be confirmed by an independent RT-qPCR assay using our previously collected lung ADC samples (c.f. Materials and Methods) (Fig. 1B). We also examined CISD2 protein expression by Immunohistochemistry (IHC) assay (Fig. 1C) using commercial lung cancer tissue microarrays. The results showed that the CISD2 protein was also significantly upregulated in lung ADC tissues (Fig. 1D). Together, these results suggest that *CISD2* expression is increased during the formation of lung ADC.

We next examined the associations of *CISD2* expression with other clinicopathological features, and found that *CISD2* mRNA expression was significantly correlated with tumor stage, grade of differentiation, or smoking status in several datasets (Supplementary Fig. S1C and D), suggesting that the transcript level of *CISD2* might also be clinically relevant to cancer progression. By performing further survival analysis using Cox’s regression model, we found that *CISD2* expression was significantly associated with prognosis of patients with lung ADC in two independent datasets. In dataset GSE8894, the *CISD2* mRNA expression level was significantly associated with recurrence-free survival (hazard ratio (HR): 2.10; 95% confidence interval (CI) of the HR: 1.21–3.64; P = 0.009). In dataset GSE31210, *CISD2* expression level was significantly associated with either recurrence-free survival (HR: 1.64, 95% CI: 1.11–2.42; P = 0.013) or overall survival (HR: 2.10, 95% CI: 1.24–3.57; P = 0.006). For each of the above datasets, when patients were stratified into two groups using the median *CISD2* mRNA expression level as a threshold, those with higher *CISD2* expression levels had significantly shorter recurrence-free survival than those with lower *CISD2* expression levels (Fig. 1E and F, P = 0.007 and P = 0.009, respectively). These data further indicate that *CISD2* might play a role in the progression of lung ADC.

### CISD2 expression positively affects cell proliferation and tumorigenicity

**Figure 2:**
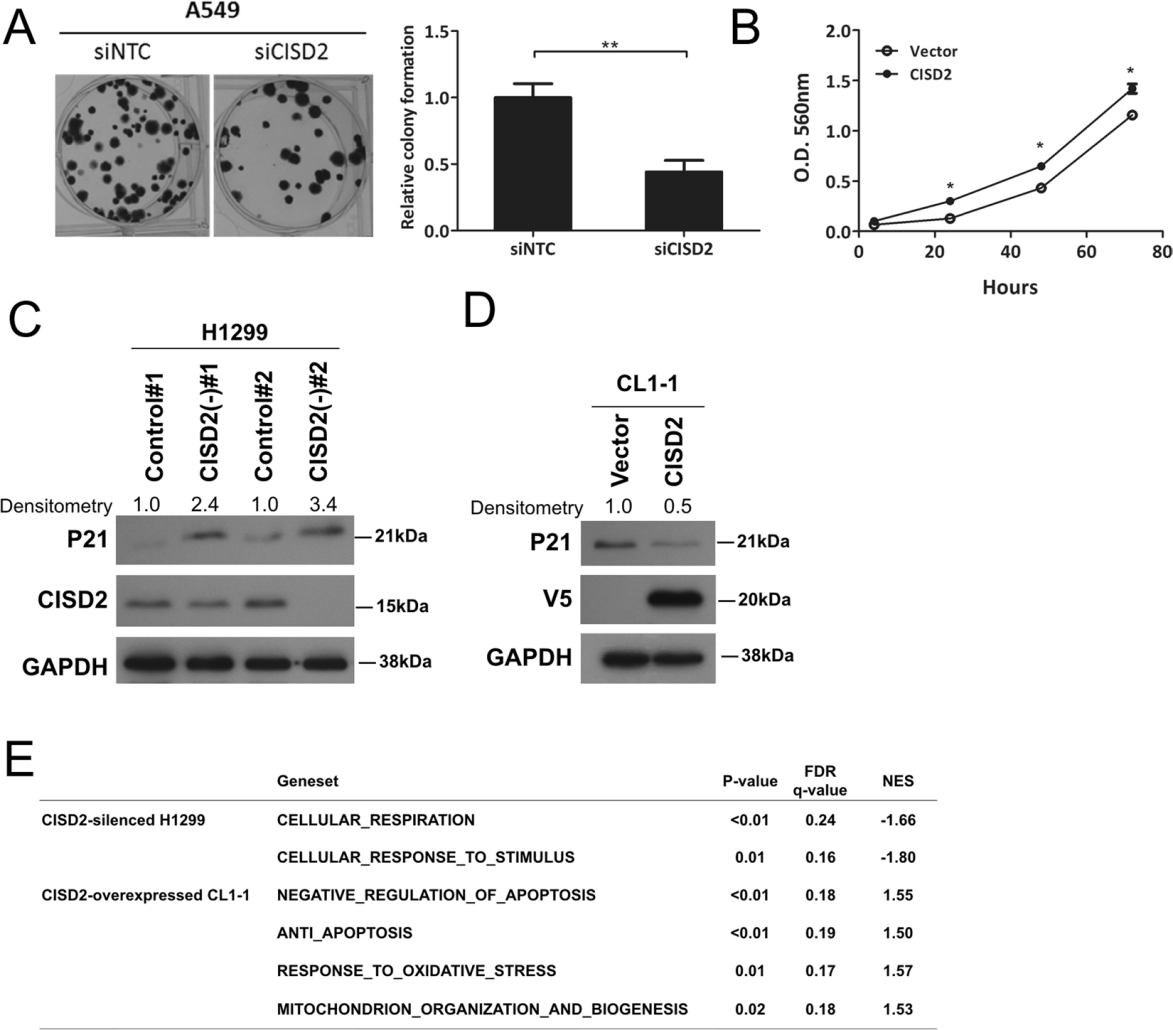
*CISD2* expression affects viability of lung ADC cells. (**A**) Left panel, representative images of clonogenic assay of si*CISD2* transfected A549 cells; right panel, result of quantification of corresponding images as in the top panel. (**B**) Viability assay of CISD2(+)-CL1-1 cells and its vector control clone. (**C** and **D**) Western blot analysis of the cell cycle regulator P21 expression in indicated cells. (**E**) GSEA analysis of CISD2 silencing or overexpression experiments in lung ADC cell lines. For (**A**) and (**B**) p values were obtained using Student’s *t*-test, *P < 0.05, **P < 0.01. All data are mean ± SD of at least triplicate measurements.

We then utilized *in vitro* cancer cell line model, in which the expression level of *CISD2* was interfered by using siRNA or shRNA techniques, to observe their effects on cancer associated phenotypes. When *CISD2* was transiently knocked down in the CISD2-abundant cell line A549 or H1299 (Supplementary Fig. S2), a significant reduction in colony formation capability, as measured by clonogenic assay, was observed (Figs 2A and S3). In addition, using CL1–1, a cell line expressing relatively low level of CISD2 (Supplementary Fig. S2), we also generated a stable transfectant overexpressing *CISD2*, which was named CISD2(+)-CL1-1. Consistently, the viability of CISD2(+)-CL1-1 was increased compared with the vector control clone when measured by MTT assay (Fig. 2B). These results suggest that *CISD2* expression is in general advantageous to the proliferation/viability or survival of lung ADC cells.

Given that *CISD2* expression might affect cancer cell proliferation, we tested whether it would influence tumor growth phenotypes *in vivo*. Compared with experiments using nontargeting control cells, when CISD2-silencing cells were injected subcutaneously into mice (n = 5 for each group), a significant retardation in the growth of xenograft tumors (Supplementary Fig. S4A and B) was observed, as was a reduction in either their mass or volume (Supplementary Fig. S4C). Due to large variation no statistical significance was established in our study, additional *in vivo* studies will be needed in order to establish the role of CISD2 in tumor development *in vivo*. Of interest, we noted that p21 (CDKN1A), an important cell cycle regulator, was significantly upregulated in two *CISD2* knockdown clones (Figs 2C and S5) and significantly downregulated in *CISD2*-overexpressing CL1-1 cells (Figs 2D and S6), implying that those CISD2-associated proliferation phenotypes might involve regulation of cell cycle progression.

We also applied bioinformatic approaches to analyze gene sets/pathways affected by forced expression or silencing of *CISD2*. We compared the differential gene expression profiles of cells having overexpressed or silenced *CISD2*, versus their own vector control cells. When these differential expression profiles were analyzed using Gene Set Enrichment Analysis (GSEA), many pathways/gene sets related to the abovementioned cellular phenotypes (Fig. 2E)—including cellular respiration, cellular responses to stimulus, anti-apoptosis, response to oxidative stress, mitochondrion organization, and biogenesis—were significantly enriched in the differential expression profiles. These consistent findings consolidate the notion that *CISD2* expression in cancer cells is associated with changes in mitochondrial function and cellular oxidative stress.

### CISD2 expression regulates cell apoptosis

**Figure 3:**
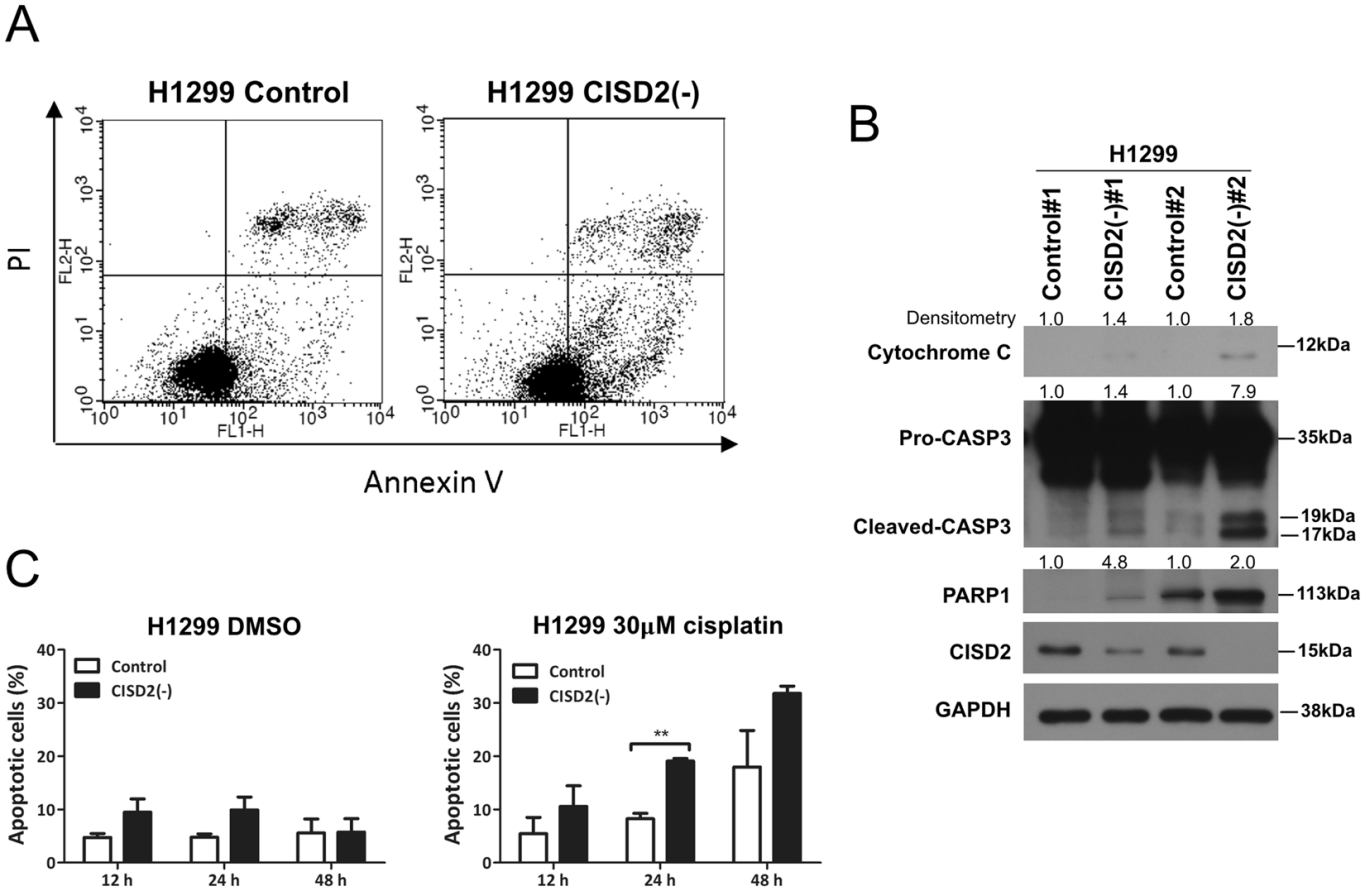
*CISD2* expression affects cell apoptosis. (**A**) Cell apoptosis assay using double staining technique. Cells were seeded overnight, and then labeled with annexin V and PI for flow cytometry analysis. (**B**) Western blot analysis of cell apoptosis markers in sh*CISD2* transfectant cells and their vector control cells. (**C**) Cell apoptosis in the absence or presence of cisplatin. Cells were seeded into six-well plates and incubated overnight, then cultured in serum-free media with or without cisplatin (30 μM). Cells were harvested at indicated time interval, labeled with annexin V and PI for flow cytometry assay. Data are mean ± SD of triplicate measurements. **P < 0.01, Student’s *t-*test.

Since our aforementioned gene expression microarray analysis indicates that CISD2 expression could affect apoptosis (Fig. 2E), we first performed annexin V/PI double staining assays. The results indicated that knocking down *CISD2* expression in lung ADC cells caused a significant increase in the apoptotic fraction (annexin V-high/PI-low; Fig. 3A). Accompanying this increase, an obvious elevation in the expression of molecular markers of apoptosis, such as cytochrome c, the cleaved form of caspase 3, and PARP1, were also detected (Figs 3B and S7) in *CISD2*-silenced cells compared with transfectants of nonsilencing shRNA. These data indicate that *CISD2* silencing is cytotoxic, which can, in turn, induce cell apoptosis. Noticeably, in the presence of the anticancer drug cisplatin, there was a doubling in the proportion of apoptotic cells in *CISD2*-depleted cells compared with the control experiment in which cisplatin was absent and no increase in the apoptotic fraction was observed (Fig. 3C, 48 h). This indicated that knocking down *CISD2* expression can enhance or sensitize lung ADC cells to the cytotoxic effects of cisplatin.

### *CISD2* expression is involved in the regulation cell invasiveness

**Figure 4:**
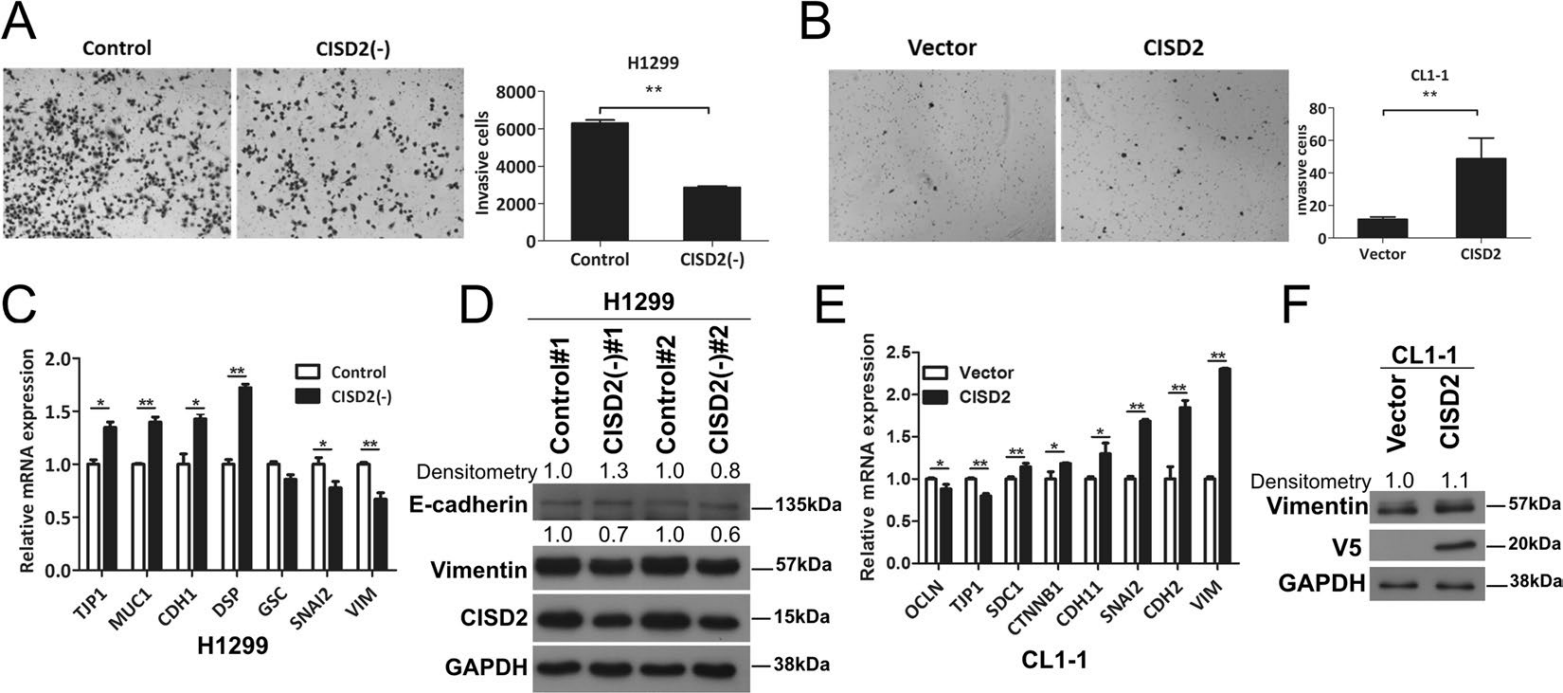
CISD2 promotes cell invasion in lung ADC cells. (**A** and **B**) Left panel, representative images of cell invasion assay showing invading cells on the bottom side of membrane of transwells; right panel, result of quantification analysis. (**C**) RT-qPCR analyses of the transcript levels of EMT markers in sh*CISD2* transfectant cells. (**D**) Western blot analysis of EMT marker proteins in sh*CISD2* transfectant cells. (**E**) RT-qPCR analyses of the transcript levels of EMT markers in CISD2(+)-CL1-1 cell. (**F**) Western blot analysis of EMT markers in CISD2(+)-CL1-1 cells. For (**A**), (**B**), (**C**) and (**E**), p values were obtained using Student’s *t*-test, *P < 0.05, **P < 0.01. All data are mean ± SD of at least triplicate measurements.

Our finding that *CISD2* expression levels is associated with patient survival implies that CISD2 could be involved in behavioral changes of cancer cells linked with cancer progression. To test this hypothesis, we conducted Transwell invasion assays to measure whether cell invasion would be affected by *CISD2* expression. Interestingly, *CISD2*-silenced H1299 cells exhibited a remarkable decrease in the number of cells that invaded (Fig. 4A), whereas forced expression of *CISD2* in CL1-1 cells resulted in a significant increase (Fig. 4B), strongly suggesting that *CISD2* expression might promote the invasiveness of lung ADC cells. To explore whether this could involve epithelial-to-mesenchymal transition (EMT), a phenomenon frequently linked to alterations in cell invasiveness, we further examined the expression of EMT markers using RT-qPCR and western blot analyses. In general, in *CISD2*-silenced H1299 cells, the expression levels of epithelial markers such as E-cadherin (*CDH1*) and desmoplakin (*DSP*) were upregulated, whereas mesenchymal markers such as vimentin (*VIM*) and zinc finger protein *SNAI2* were downregulated (Figs 4C,D and S8). By contrast, in *CISD2*-overexpressing cells, epithelial markers such as tight junction protein (*TJP*)-1 (also known as zona occludens 1 or ZO1) were downregulated, whereas mesenchymal markers such as *VIM*, *CDH2*, and *SNAI2* were significantly upregulated (Figs 4E,F and S9). These data indicate that EMT might be involved in the *CISD2*-induced increase in cell invasion potential.

### CISD2 contributes to the maintenance of mitochondrial function and the suppression of ROS production in lung cancer cells

**Figure 5:**
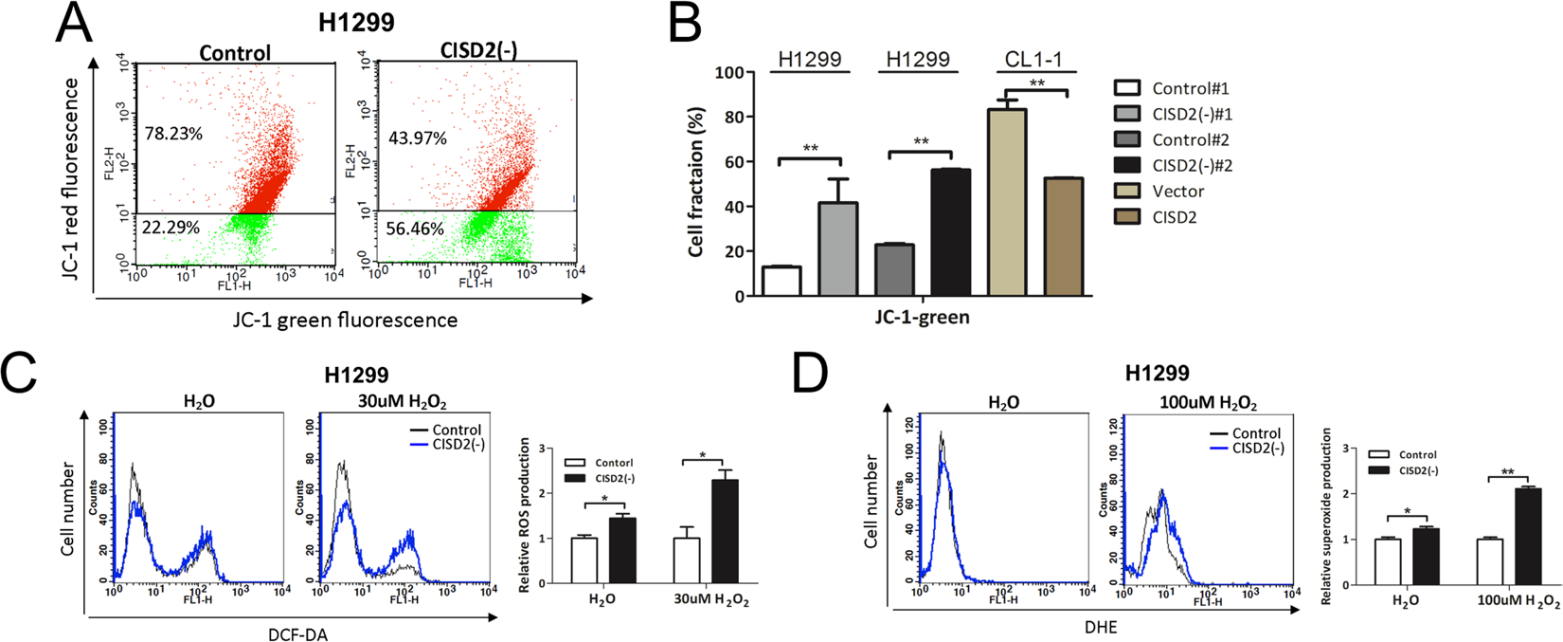
Silencing of *CISD2* causes dysfunction of mitochondria in lung ADC cells. (**A**) Flow cytometry assay for detection of change in mitochondrial membrane potential using JC-1 dye. (**B**) JC-1 green fluorescence quantification analyses of experiments of silencing or overexpressing *CISD2* in lung ADC cell lines. (**C** and **D**) Measurements of ROS levels using DCFH-DA and DHE dyes. CISD2(−)#1-H1299 cells were seeded overnight, refreshed in serum free medium in the absence (left panels) or presences of indicated amount of H_2_O_2_ (middle panels) for 20 m, then subjected to flow cytometry assay. Right panels, results of quantification. For (**B**), (**C**) and (**D**), data are mean ± SD of triplicate measurements. *P < 0.05, **P < 0.01, Student’s *t-*test.

Previous studies suggested that CISD2 plays a role in maintaining mitochondrial integrity and respiration capacity^11,13^. Our GSEA analysis of microarray data also identified involvement of mitochondrial, oxidative stress, and ROS signaling gene sets or pathways in *CISD2-*silencing or *CISD2-*overexpressing cell lines (Fig. 2E). We therefore hypothesized that the oncogenic features of CISD2 involve its roles in the maintenance of mitochondrial function in lung ADC cells. To test this, we utilized flow cytometry to detect whether mitochondrial membrane potential was affected in *CISD2* silencing context. As shown in Fig. 5A and B, a 34% ± 0.7% increase in the fluorescence intensity of the cationic dye JC-1 was observed in *CISD2* silencing conditions, indicating a significant drop in the mitochondrial membrane potential. By contrast, a 31% ± 4.2% decrease in JC-1 fluorescence intensity was found in *CISD2* forced expression conditions (Fig. 5B). These data strengthen our conjecture that CISD2 might play a critical role in mitochondrial homeostasis in lung ADC cells.

Because mitochondria are one of the major sources of ROS in cells, it is possible that imbalanced homeostasis or damage of mitochondria might result in the release of ROS, leading to oxidative damage and impairing the survival of cancer cells. Therefore, it would be of interest to know whether targeting *CISD2* expression could induce the generation of ROS. To address this, we used the redox-sensitive dyes 2′,7′-dichlorofluorescein diacetate (DCFH-DA) and dihydroethidine (DHE) to measure the levels of intracellular ROS and superoxide radicals, respectively. We found that the fluorescence intensities of both DCF and DHE were significantly increased in *CISD2*-silenced H1299 cells CISD2(−)#1, compared with control cells (left panels of Fig. 5C and D), indicating that CISD2 depletion leads to the accumulation of ROS and superoxide radicals in cells. Markedly, such accumulation became even more obvious in the additional presence of hydrogen peroxide (right panels of Fig. 5C and D), suggesting that under stress conditions, CISD2 is required to an even greater degree for maintenance of ROS homeostasis in lung ADC cells. These consistent findings consolidate the notion that *CISD2* expression in lung ADC is associated with changes in mitochondrial function and cellular oxidative stress.

### Downregulation of CISD2 induces expression of antioxidant GPX3

**Figure 6:**
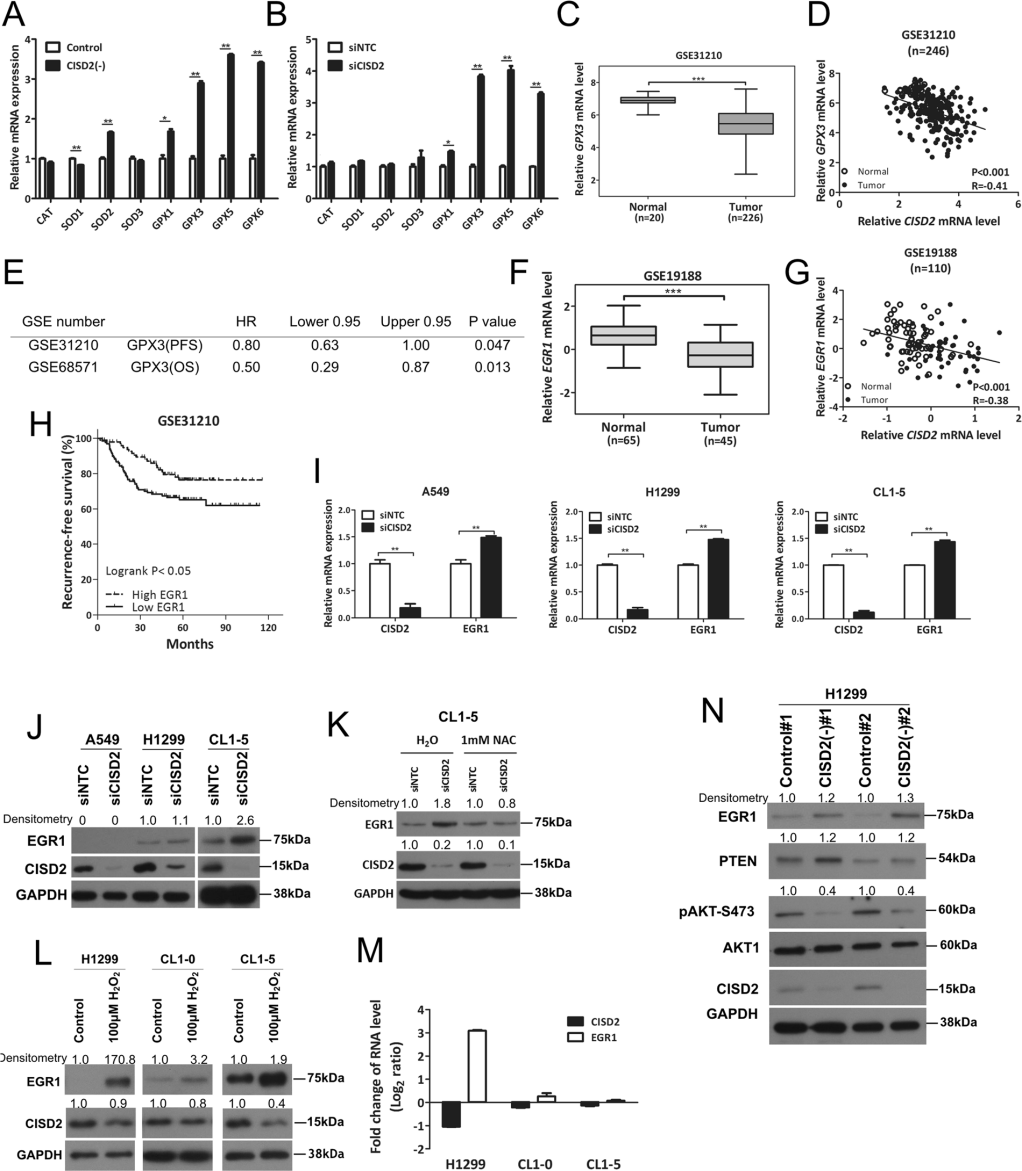
CISD2 negatively regulates mRNA expression of antioxidant gene *GPX3* and ROS-mediated EGR1 signaling. (**A** and **B**) RT-qPCR analysis of expression levels of selected antioxidant genes using sh*CISD2* transfectant CISD2(−)#1-H1299 (**A**) or si*CISD2* transfected cells harvested at 48 h post transfection (**B**). (**C**) *GPX3* mRNA expression profile of lung ADC *versus* normal lung tissues extracted from public domain dataset GSE31210. (**D**) Scatter plot showing significantly negative correlation between mRNA expression levels of *CISD2* and *GPX3* in public dataset GSE31210. Correlation test was used to estimate the significance of correlation. (**E**) Cox’s regression analyses (age adjusted) of *GPX3* mRNA expression and prognostic outcome in patients with lung ADC. PFS, progression-free survival; OS, overall survival. (**F**) *EGR1* mRNA expression profile of lung ADC *versus* normal lung tissues extracted from public domain dataset GSE19188. (**G**) Scatter plots showing significant negative correlation between mRNA expression levels of *CISD2* and *EGR1* in public dataset GSE19188. (**H**) Survival analysis on *EGR1* mRNA expression and recurrence-free survival using public domain data GSE31210. Patients were stratified into low EGR1 (solid line) and high EGR1 (dashed line) groups using median *EGR1* expression level as cutoff; significance of difference in survival between groups was estimated using log rank test. (**I**) *EGR1* mRNA expression in si*CISD2* transfected lung ADC cell lines as measured by RT-qPCR assay. Cells were harvested at 48 h post transfection. (**J**) Western blot of CISD2 and EGR1 proteins of cells used in (**I**). (**K**) Western blot analysis of CISD2 silencing-induced upregulation of EGR1 in CL1-5 cells was reduced when cells were pretreated with 1 mM of NAC for 1 h before siCISD2 transfection. (**L** and **M**) Western blotting (**L**) and RT-qPCR (**M**) analyses of *CISD2* and *EGR1* expression in H1299, CL1-0 and CL1-5 cells treated with 100 μM H_2_O_2_ for 1 h. (**N**) Western blotting of putative targets downstream EGR1 signaling in CISD2 silencing clones. For (**A**), (**B**) (**C**), (**F**) and (**I**), p values were obtained using Student’s *t*-test, *P < 0.05, **P < 0.01, ***P < 0.001. All data are mean ± SD of at least triplicate measurements.

In response to increases in ROS accumulation upon perturbation caused by CISD2 depletion, the detection of antioxidant or stress response gene expression would be expected. In this regard, we noted that some genes encoding antioxidant enzymes such as *SOD2*, *GPX1*, *GPX3*, *GPX5*, and *GPX6* showed markedly elevated expression levels when *CISD2* was silenced in H1299 cells, as evidenced by RT-qPCR profiling (Fig. 6A and B). Among those genes, *GPX3*, which encodes a glutathione peroxidase, an antioxidant enzyme and putative tumor suppressor that defends cells against ROS, was the most significantly upregulated. This suggests that knocking down *CISD2* might cause upregulation of *GPX3* and further implies that *CISD2* expression may inhibit *GPX3* expression. In support of this, from analyses of public data derived from clinical lung ADC samples, we found that not only was *GPX3* expression significantly downregulated in several independent datasets (Figs 6C and S10A), but also there was a significant negative correlation between the mRNA levels of *CISD2* and *GPX3* in related datasets (Figs 6D and S10B). Furthermore, since CISD2 was shown in a previous session to be positively associated with poor prognosis, it is of interest to find that *GPX3* expression exhibited a significantly negative correlation with poor prognosis in two independent datasets (Fig. 6E). Jointly, these *in vitro* and clinical samples-based data suggest that upregulation of *CISD2* might contribute to carcinogenesis and/or cancer progression by regulating ROS homeostasis, and, therefore, offer cells a survival advantage by protecting them from raised oxidative stress, which in turn prevents the production of antioxidant genes, such as *GPX3*.

### CISD2 silencing-induced EGR1 expression is mediated via ROS and affects AKT signaling

By the same token as in the case of CISD2-GPX3 axis, we hypothesized that upregulation of *CISD2* expression in lung ADC cells might prevent the expression of tumor suppressors associated with ROS/oxidative stress response. To test this possibility, we examined the correlation between *CISD2* expression and known stress response genes. Among others, we found that *EGR1* expression is not only downregulated in many lung ADC datasets (Figs 6F and S10C), but also significantly negatively correlated with *CISD2* expression in multiple datasets (Figs 6G and S10D). Again, since that *CISD2* expression is associated with poor prognosis and that *CISD2* and *EGR1* mRNA levels are negatively correlated, we anticipated that EGR1 might be a favorable prognostic factor in the context of lung ADC. As expected, we found that *EGR1* expression was positively correlated with either recurrence-free patient survival (Fig. 6H) or overall survival (Supplementary Fig. S10E).

We resorted to cell-based assays again to find possible supporting evidence. Consistent with these clinical sample-based observations, we found that *EGR1* expression can be transcriptionally affected by *CISD2* expression, as *CISD2* silencing triggered *EGR1* mRNA (Fig. 6I), no matter EGR1 protein was detectable (as for H1299 and CL1-5 cell lines, Figs 6J and S11) or not (as for A549 cell line, Figs 6J and S11), indicating that CISD2 could repress *EGR1* expression. We noted that EGR1 protein expression was undetectable in A549 cells is also consistent to an earlier report by Zhang *et al*.^21^; this suggests that in addition to regulation by CISD2, there might exist other unidentified pathway(s) involved in the suppression of EGR1 protein expression. However, when EGR1 protein expression was detectable, silencing of *CISD2* expression can also cause a moderate to strong upregulation of EGR1 protein expression (Figs 6J and S11).

To test our conjecture that the negatively correlated expression levels between *CISD2* and *EGR1* might be mediated by ROS, we performed two experiments. In the first, we examined whether the upregulation of *EGR1* upon *CISD2* silencing could be attenuated in the presence of a ROS scavenger. As shown in Figs 6K and S12, in the presence of N-acetyl-l-cysteine (NAC), there was no increase in EGR1 protein level upon silencing of *CISD2*, confirming that the upregulation of EGR1 expression requires ROS. In the second, we explored whether *EGR1* or *CISD2* expression level would be affected by treatment with hydrogen peroxide in lung ADC cells. As expected, the expression of EGR1 was upregulated significantly (Figs 6L and S13). Intriguingly, levels of the *CISD2* protein (Figs 6L and S13) or its mRNA (Fig. 6M) were both downregulated and also seemed inversely correlated with those of *EGR1* among the three cell lines used, again confirming that ROS mediates CISD2–EGR1 signaling.

As *EGR1* is responsive to ROS-mediated signaling from *CISD2*, we explored whether the downstream signaling of EGR1 might be affected by *CISD2* expression. Interestingly, the protein expression of phosphatase and tensin homolog (PTEN), reported as a downstream factor of EGR1^22,23^, was significantly enhanced in lung ADC cells (Figs 6N and S14). Closely associated with and perhaps attributed to the increase in PTEN, the level of phosphorylation of AKT at serine residue 473 (S473) was significantly attenuated (Figs 6N and S14) as expected. Collectively, these results imply that CISD2 may function as an oncogene by regulating ROS homeostasis and EGR1 induction, which further dysregulate PTEN/AKT signaling in lung ADC cells.

## Discussion

**Figure 7:**
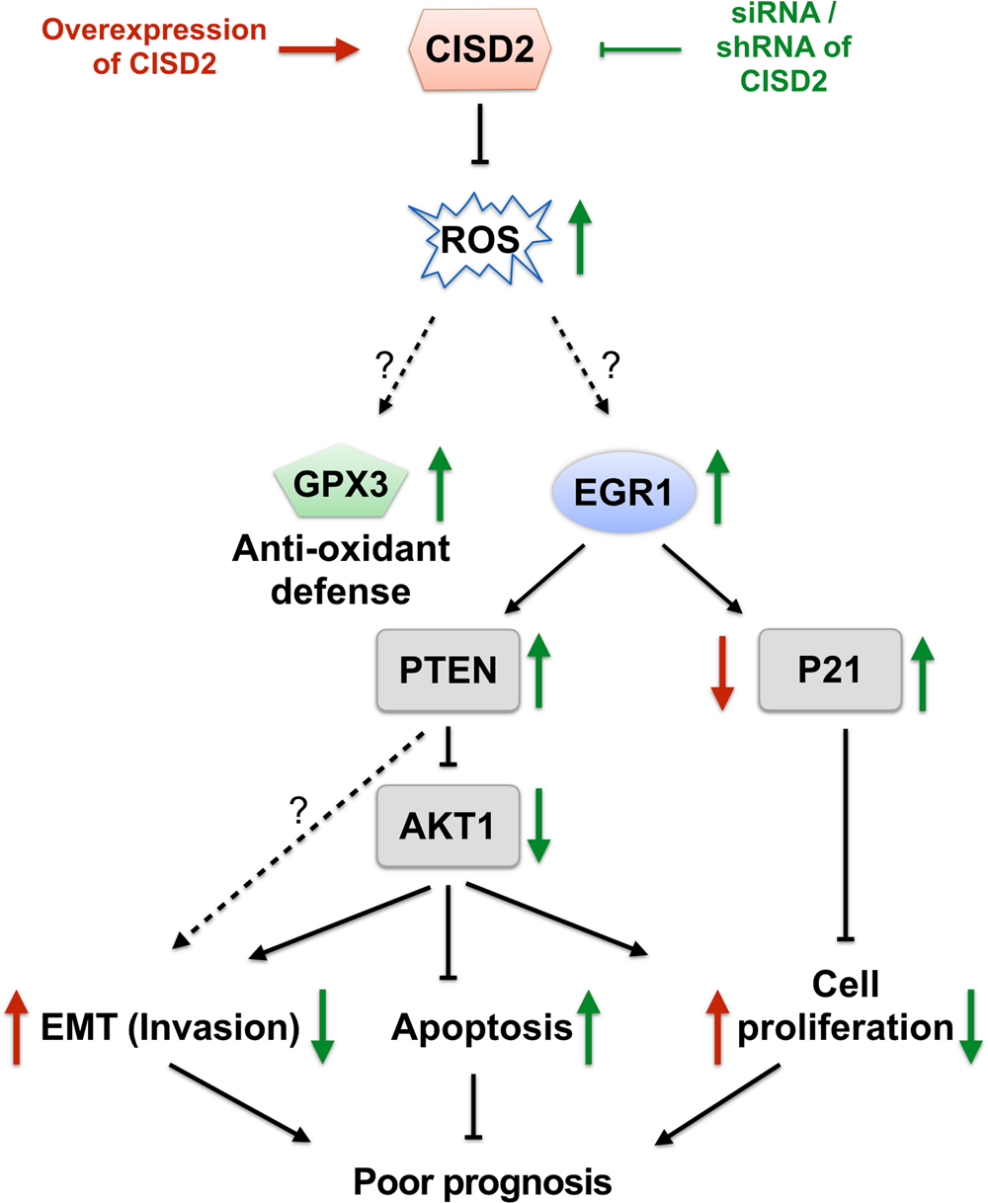
A proposed model relating CISD2, GPX3 and EGR1 signaling in lung ADC cells to poor prognosis of lung ADC patients. Texts/arrows in red represent observations from cell based assays under *CISD2* overexpressed condition; texts/arrows in green represent those under *CISD2* silencing condition. Arrows pointing upward, upregulation; arrows pointing downward, downregulation.

In this study, using a combined approach relating evidence from analysis of public gene expression data derived from clinical specimens of lung cancer tissues, cell-based assays, and animal xenograft models, we have identified the oncogenic properties of CISD2 and its clinical significance in lung ADC. To the best of our knowledge, this is the first report to link CISD2 with lung cancer. In addition to establishing an association of important clinical relevance, we also provide some possible molecular explanations of how the upregulation of *CISD2* expression could foster cancer cell formation and progression. We propose a model of CISD2–ROS–EGR1/GPX3 axis signaling (Fig. 7), in which increased levels of CISD2 contribute to the neutralization of excessive ROS production, which would otherwise increase antitumor activity mediated at least by the tumor suppressors EGR1 and GPX3. The oncogene nature of *CISD2* revealed here could be analogous to that of the NRF2–KEAP1 system in lung cancer, where NRF2, a well-known stress response gene hijacked by cancer cells^24^, was found to be significantly associated with prognosis^25^.

CISD2 belongs to the CDGH iron sulfur domain-containing protein family, which contains a 2Fe-2S cluster known to function as an active redox-sensitive center^26,27^. Other proteins that contain a 2Fe-2S cluster are also known to have important roles in redox transition^26^. Given that CISD2 is a redox-sensitive protein localized in mitochondria^11,28,29^ and the ER^12,30^, which are two major cellular sources of ROS, and that depletion of CISD2 in lung cancer cells can lead to the accumulation of ROS as shown in our data, it is highly plausible that CISD2 is an antioxidant protein capable of reducing ROS to maintain redox homeostasis in cancer cells. Therefore, a reasonable scenario emerges: during the process of cancer formation or progression, the upregulation of CISD2 offers cancer cells critical survival and proliferation signals by providing them with greater antioxidant capacity to counteract gradually elevated ROS levels. In such a scenario, the expression level of *CISD2* could be a marker reflecting the levels of accumulated oxidative stress in cancer cells. This could explain why the *CISD2* level is significantly higher in lung ADC patients who are smokers than in those who are nonsmokers (Supplementary Fig. S1D), reflecting exposure to ROS generated exogenously. It could also explain why the *CISD2* level is positively associated with the clinical stage and grade of differentiation (Supplementary Fig. S1D), reflecting an elevated overall ROS level during cancer progression. In the emerging milieu, where targeting the antioxidant capacity of tumor cells could have a positive therapeutic impact^31^, further investigations are needed into the biochemical properties of CISD2, its interacting partners, and how it is regulated in cancer cells.

Our finding that CISD2 silencing-induced ROS can promote the expression of putative tumor suppressor EGR1 is also novel to lung cancer biology. *EGR1* is known as a stress-response gene that plays a protective role when cells suffer starvation, ultraviolet light irradiation, hypoxia, and oxidative stress. It functions as a tumor suppressor gene in various types of cancer by inducing cell cycle arrest and apoptosis^32–34^ and by promoting other potent tumor suppressors such as P53^22^, PTEN^22,23^, and p21 CDK1A^32,35^. In a colon cancer cell model, drug-induced apoptosis has been shown to be mediated by ROS–EGR1 signaling^36^. More relevant to lung cancer, EGR1 expression can predict the expression of its putative downstream target PTEN^37^; this is also in agreement with our data. We showed that knocking down *CISD2* expression significantly increased EGR1 and PTEN protein levels, and eventually decreased the level of phospho-AKT (Figs 6N and S14). A similar mechanism may also act in gastric cancer cells, in which CISD2 positively regulates AKT activity^16^. Downregulation of EGR1 has been implicated in the changes of mobility and migratory ability of lung cancer cells^21^, or in the process of EMT^38^. Furthermore, EGR1/PTEN/AKT axis was known to play a key role in inhibiting EMT through downregulation of AKT phosphorylation^39,40^. Since EMT phenotype observed in cell based study could provide a much likely link to poor prognosis of patients in clinical setting, our findings that CISD2 may regulate EGR1 expression and, at the same time, positively affect EMT phenotype (Fig. 4) become very important. It would be useful to know whether similar phenotypes that are affected by CISD2 are mediated through ERG1.

Overall, we found that the upregulation of *CISD2* expression was involved in the tight regulation of redox homeostasis, which fosters the development and progression of lung ADC by enabling escape from apoptosis and the gain of cell proliferation and invasion potential. These findings strongly suggest that it would be worth investigating whether CISD2 or its associated downstream targets can be considered when developing anticancer therapies. For instance, because knocking down *CISD2* expression or targeting CISD2 with pioglitazone, as exemplified by Darash-Yahana and colleagues^14^, can lead to an imbalance in ROS homeostasis and an increase in oxidative stress, methods for inhibiting the action of CISD2 may be developed to assist in the eradication of lung cancer cells, or to function as sensitizers to enhance chemotherapy or radiotherapy, in a similar way to that proposed for the NRF2–KEAP1 scenario in lung cancer treatment^41,42^.

Although this study has focused on lung ADC in terms of the oncogenic role of the CISD2–ROS–EGR1/GPX3 axis, we found that *CISD2* expression was also upregulated and seemed to be inversely correlated with prognosis among patients with lung SQC (unpublished data), another major lung cancer subtype that is etiologically and genetically distinct from lung ADC. It would be worth investigating whether CISD2 also plays an oncogenic role in lung SQC and to explore its underlying mechanisms for identification of therapeutic targets in corresponding pathways for the effective treatment of lung cancers.

## Materials and Methods

### Public domain data

Several lung cancer datasets, including GSE31210^18^, GSE27262^19^, GSE19188^20^, GSE8894^43^, and GSE10245^44^ were downloaded from Oncomine (http://www.oncomine.com). In addition to the above datasets generated by others, our in-house-generated dataset GSE46539^45^ has been deposited in the Gene Expression Omnibus (https://www.ncbi.nlm.nih.gov/geo/) and was also utilized in this study. Information about the probes of genes of interest applied in this study can be found in Supplementary Table S1. For each dataset, gene expression data and associated clinical information of samples belonging to cancer subtype of lung ADC or normal lung tissues were all downloaded without selection. Since data from Oncomine have already been normalized, they were used directly without further processing.

### Clinical samples

Fifty-six clinical lung tissue samples, which comprise 28 normal lung samples and 28 lung ADC samples, originated from a cohort we have described previously^46^. These samples were obtained from surgical resection samples collected at Chang Gung Memorial Hospital, Taoyuan, with approval from the Institutional Review Board of Chang Gung Memorial Hospital and informed consent from all participants. All procedures and methods were achieved in accordance with the relevant guidelines and regulations. Human lung cancer tissue microarrays LC810 and LC1006 were purchased from US Biomax (Rockville, MD, USA); CCA3, CC4, and CCN4 were from Super Bio Chips (Seoul, Korea). Collectively, these arrays contain 47 lung ADC and seven normal lung tissues. Corresponding clinical information of each specimen was provided by the manufacturers.

### Cell lines and DNA constructs

Lung ADC cell lines NCI-H1299 and A549 were obtained from American Type Culture Collection (ATCC, Manassas, VA, USA). CL1-1 and CL1-5 lung ADC cell lines were gifts from Dr. Cheng-Wen Wu as described previously^47^. All cell lines were maintained in RPMI 1640 medium (Invitrogen, Grand Island, NY, USA) supplemented with 10% FBS (Invitrogen) and 1% penicillin/streptomycin (Biological Industries, Cromwell, CT, USA). To construct a plasmid expressing the full-length *CISD2*, the coding sequence of *CISD2* in an EST clone (IMAGE:5105935) was amplified by PCR with the forward primer 5′-GGATCCATGGTGCTGGAGA-3′ and reverse primer 5′-GAATTCTTATACTTCTTTCTTCTTCAGT-3′ and then subcloned into a pcDNA3.1 expression vector (Invitrogen). Cell transfection was conducted with Lipofectamine 2000 (Invitrogen) and cells were maintained in selection media containing 2 mg/mL G418 (Calbiochem, San Diego, CA, USA).

### siRNA

An siRNA mixture (SMARTpool) targeting human *CISD2* gene expression containing the following sequences: 5′-UCAGAAUGGCUUCGGUUAU-3′, 5′-UAUUGUAGGUGUUGGCGUU-3′, 5′-CCUGAAAGCAUUACCGGGU-3′, and 5′-UGGAGAGCGUGGCCCGUAU-3′, was obtained from Dharmacon (GE Healthcare, Pittsburg, PA, USA). The negative control siRNAs (nontargeting pool; Dharmacon) were used according to the manufacturer’s instructions.

### shRNA constructs

The shRNA expression vectors targeting *CISD2* (CISD2(−)#1 and CISD2(−)#2) were purchased from RNAi Core Lab (Academia Sinica, Taipei, Taiwan) and transOMIC (Huntsville, AL, USA), respectively, as well as their negative controls. Plasmid DNA was transfected into cells according to the manufacturer’s instructions. Cells were maintained in selection media containing 2 μg/mL puromycin (Calbiochem).

### GSEA

To analyze biological pathways affected by silencing or forced ectopic expression of *CISD2* in lung ADC cell lines, the gene expression profiles of CISD2-silencing H1299 cells and CISD2-overexpressed CL1-1 cells and their nontargeting or vector control cells were obtained by performing Illumina HT12 Beadchip experiments as previously described^46^. GSEA^48^ was then used to obtain gene sets or biological pathways enriched in the differential expression profiles using log_2_R as ranking metric, where R was the ratio of normalized gene expression level of a gene in test cells to that in control cells. GSEA Desktop Application (http://software.broadinstitute.org/gsea/downloads.jsp) was used for GSEA implementation.

### RT-qPCR

Total RNA was reserve-transcribed by using SuperScript III (Invitrogen) and oligo(dT) primers per the manufacturer’s instructions. Methods for RT-qPCR and analysis for the quantification of relative mRNA level were as described previously^47^. The primers and the Universal Probe Library probes (Roche Molecular Diagnostics, Pleasanton, CA, USA) are listed in Supplementary Table S2.

### IHC analysis

IHC staining was performed using rabbit anti-CISD2 antibodies (Sigma-Aldrich, St Louis, MO, USA, HPA015914, 1:25) following a procedure described previously^49^. The immunoreactivity patterns of all tissues were scored by two independent pathologists. Staining intensity was scored as 0 (negative), 1+ (weak), 2+ (moderate), and 3+ (strong), as described elsewhere^46^.

### Immunoblot analysis

Protein separation and western blotting protocols were as described previously^47^. Briefly, cells were lysed with RIPA solution, succeeded to SDS-PAGE gel electrophoresis, and the proteins transferred to PVDF membranes. The proteins were probed with anti-CISD2 (1:1,000 dilution; HPA015914, Sigma-Aldrich), anti-V5 (1:2,000 dilution; R960, Invitrogen), anti-GAPDH (1:10,000 dilution; MAB374, Millipore, Billerica, MA, USA), anti-E-cadherin (1:1,000 dilution; #3195, Cell Signaling Technology), anti-vimentin (1:1,000 dilution; #5741,Cell Signaling Technology), anti-ZO-1 (1:1,000 dilution; #5406, Cell Signaling Technology), anti-P21 (1:1,000 dilution; #2947,Cell Signaling Technology), anti-cytochrome C (1:1,000 dilution; GTX108585, GeneTex), anti-caspase 3 (1:1,000 dilution; GTX110543, GeneTex), anti-PARP1 (1:1,000 dilution; GTX100573, GeneTex), anti-EGR1 (1:1,000 dilution; #4153, Cell Signaling Technology), anti-PTEN (1:1,000 dilution; #9188, Cell Signaling Technology), anti-phospho-AKT Ser473 (1:1,000 dilution; (#4060, Cell Signaling Technology), and anti-AKT1 (1:1,000 dilution; #2938, Cell Signaling Technology) antibodies, and then further probed by horseradish peroxidase-conjugated secondary antibodies.

### Cell viability assay

Cells (~2 × 10^4^) were seeded in 24-well plates for predetermined time intervals. The protocol for MTT assays was as described previously^47^.

### Clonogenic assay

Serially diluted cells (~1 × 10^2^) were seeded with culture medium in six-well plates and cultured for 8 to 12 days. Colonies were stained with 1% crystal violet (Merck, Darmstadt, Germany) and at least 50 cells were counted.

### Animal experiments

For xenograft tumor experiments, a total of 1 × 10^6^ cells in 100 μL PBS were injected subcutaneously into five severe combined immunodeficient mice (BALB/cAnN.Cg-Foxn1nu/CrlNarl) for each test (sh*CISD2*) and control (nontargeting shRNA) groups. The volume of tumor formed was measured over a 7-week period post-injection. Tumor volume was calculated according to the formula of length × width × width/2. Tumor mass was evaluated upon sacrifice at the seventh week after injection. All experimental procedures of the animal use protocol was approved by the Institutional Animal Care and Use Committee of National Health Research Institutes (NHRI) and all methods were performed in accordance with the relevant guidelines and regulations.

### Apoptosis assay

siRNA-treated cells or stable transfectants of sh*CISD2* were seeded into six-well plates and incubated overnight. Culture media were replaced by a serum-free medium with or without cisplatin (30 μM), and cells were harvested at 12, 24 and 48 h, labeled with annexin V and PI (Invitrogen) per the manufacturer’s instructions, and then analyzed by flow cytometry.

### Invasion assay

siRNA-treated cells or stable transfectants of sh*CISD2* in serum-free media were seeded into a cell culture insert (Falcon, Corning, NY, USA) at a density of 1 × 10^4^/well and placed into a 24-well plate with regular medium. After incubation for 16 h, Matrigel (BD Biosciences) and cells remaining on the seeding side of the membrane were wiped off with cotton swabs, while cells that invaded the bottom surface of the membrane were fixed with methanol (Merck) and detected using Giemsa stain (Sigma-Aldrich).

### Mitochondrial membrane potential assay

Cells were seeded into six-well plates and incubated for 24 h, then transferred to serum-free medium containing 2 μg/mL JC-1 (eBioscience, San Diego, CA, USA), a membrane potential-sensitive cationic dye, incubated at 37 °C for 20 min, washed with PBS, and then analyzed using flow cytometry. Aggregated JC-1 exhibits red fluorescence, indicating a high membrane potential, while monomer JC-1 shows green fluorescence, which indicates a membrane potential collapse.

### Measurement of intracellular ROS

To detect the production of intracellular ROS, cell membrane-permeable fluorogenic probes DCFH-DA (Sigma-Aldrich) and DHE (Sigma-Aldrich) were applied. Cells were seeded into six-well plates and incubated for 24 h, and transferred into serum-free medium containing 10 μM DCFH-DA or 10 μM DHE. After incubation at 37 °C for 20 min, cells were harvested and washed with PBS, and then analyzed using flow cytometry.

### Statistical analysis

Student’s *t*-test was used to assess the significance of difference in mRNA or protein levels between conditions of interest. Cox proportional hazard regression model was used to analyze the association between mRNA expression level and patients’ survival. The log-rank test was used to evaluate the significance of difference in survival between patients of stratified groups. Pearson correlation analysis was used to assess the significance of correlation between mRNA expression levels of a pair of genes of interest. In all analyses, P < 0.05 was considered statistically significant.

## Electronic supplementary material

Supplementary information
