# Supplementary material for: Upregulation of CISD2 augments ROS homeostasis and contributes to tumorigenesis and poor prognosis of lung adenocarcinoma
Source: Sci Rep. 2017 Sep 19;7:11893. doi: 10.1038/s41598-017-12131-x (PMC5605537; doi:10.1038/s41598-017-12131-x)
Supplement: Supplementary file 1 — Supplementary information [file 41598_2017_12131_MOESM1_ESM.pdf]

Supplementary information

Upregulation of *CISD2* augments ROS homeostasis and contributes to tumorigenesis and poor prognosis of lung adenocarcinoma

Shih-Miao Li [1,2,3], Chung-Hsing Chen [3], Ya-Wen Chen [3], Yi-Chen Yen [3], Wen-Tsen Fang [3], Fang-Yu Tsai [3], Junn-Liang Chang [4,5,] , Ying-Ying Shen [6], Shiu-Feng Huang [7], Chih-Pin Chuu [8,9], I-Shou Chang [3], Chao A. Hsiung [1,2*], and Shih Sheng Jiang [3*]

[1] Institute of Bioinformatics and Structural Biology, National Tsing Hua University, Hsinchu, Taiwan

[2] Institute of Population Health Sciences, National Health Research Institutes, Miaoli, Taiwan

[3] National Institute of Cancer Research, National Health Research Institutes, Miaoli, Taiwan.

[4] Department of Pathology & Laboratory Medicine, Taoyuan Armed Forces General Hospital, Taoyuan, Taiwan

[5] Biomedical Engineering Department, Ming Chuang University, Taipei, Taiwan

[6] Pathology Core Laboratory, National Health Research Institutes, Miaoli, Taiwan

[7] Institute of Molecular and Genomic Medicine, National Health Research Institutes, Miaoli, Taiwan

[8] Institute of Cellular and System Medicine, National Health Research Institutes, Miaoli County, Taiwan

[9] Graduate Program for Aging, China Medical University, Taichung City, Taiwan

*Correspondence should be addressed to C.A.H. (address: 35 Keyan Road, Zhunan, Miaoli County 35053, Taiwan, hsiung@nhri.org.tw, tel.: +886-37-246-166 ext. 36100, fax: +886-37-586-467), and to S.S.J (address: 35 Keyan Road, Zhunan, Miaoli County 35053, Taiwan, ssjiang@nhri.org.tw, tel.: +886-37-246-166 ext. 31708, fax: +886-37-586-463)

Supplementary Table S1. Public domain gene expression microarray datasets used in this study and probes information

	GSE31210	GSE27262	GSE19188	GSE8894	GSE10245	GSE46539	GSE32863	GSE68571
Case no.	246	50	110	63	40	115	116	96
ADC	226	25	45	63	40	115	58	86
Normal	20	25	65	0	0	115	58	10
Matched pair	No	No	No	No	No	Yes	No	No
Platform	Human Genome U133 Plus 2.0 Array	Human Genome U133 Plus 2.0 Array	Human Genome U133 Plus 2.0 Array	Human Genome U133 Plus 2.0 Array	Human Genome U133 Plus 2.0 Array	Illumina HumanHT-12 WG-DASL V4.0 R2 expression beadchip	Illumina HumanWG-6 v3.0 Expression Beadchip	Affymetrix Human Full Length HuGeneFL Array
Probe ID								
CISD2	226686_at	226686_at	226686_at	226686_at	226686_at	ILMN_1796397	ILMN_1796397	-
GPX3	201348_at	201348_at	201348_at	201348_at	201348_at	-	ILMN_1726666	D00632_at
EGR1	201693_s_at	201693_s_at	201693_s_at	201693_s_at	201693_s_at	-	ILMN_1762899	X52541_at
Source	Oncomine	Oncomine	Oncomine	Oncomine	Oncomine	In-house generated	Oncomine	Oncomine

Supplementary Table S2. Primers used in RT-qPCR

Gene	Forward primes	Reverse primers	Probe no.
CISD2	CCTTGTATACGCTAGGGACTGG	GGGACTTGAGTATGGGCAGA	81
TJP1	AAATTTAACTAATGTCAGACTGGAGGA	TCAGCTTGTGGTGAGTAAGAGG	17
MUC1	GGCAGCAGCCTCTCTTACAC	CCCCTACAAGTTGGCAGAAG	3
CDH1	CAGGCTCAAGCTATCCTTGC	AGTCATGCGTAGTGGTGCAT	33
DSP	GAGCCTTCTGGTGAAAATCAA	ACGATTCAGCTCATCCTCCA	1
GSC	CGCTCTCTTTCGGTTTGGT	CGAGGACAGAGCCTTAAAGTG	19
SNAI2	CCATGCCTGTCATACCACAA	ACAGTGATGGGGCTGTATGC	73
VIM	TTCGCCAACTACATCGACAA	CGGCCAGCAGGATCTTATT	1
OCLN	CCGAGTTTCAGGTGAATTGG	GGGAGTGTAGGTGTGGTGTGT	52
SDC1	TTCCAGGGTCCCTCTGTGTA	ACCTTGGCTGAACCTACCG	2
CTNNB1	TGTTAAATTCTTGGCTATTACGACA	TTGCTTTCTTGGTTGCCATA	8
CDH2	ACGCTCTCCCTCCCTGTT	GGACTCGCACCAGGAGTAAT	17
CAT	TAACCCGCTCATCACTGGAT	GAAGCCACTAGCTTGCATTTG	2
SOD1	AGATGACTTGGGCAAAGGTG	TATTGGGCGATCCCAATTAC	11
SOD2	GCACTAGCAGCATGTTGAGC	GAGCCCAGATACCCCAAAAC	27
SOD3	TGCTTTTCCTCCCTGAACTG	AGCCCAGAGGAGTGGTCAG	12
GPX1	GTGCTGGTCCTGTTGATCC	CTGACACCCGGCACTTTATT	53
GPX3	GGGAGCTGAGGGCAAGTC	GAGACCCTTGCAGCCAATC	28
GPX5	CCCATCTTCTCACCACACTCT	GCAGTATGTGGCGGTGATTT	39
GPX6	CCTCCTTCTTTGCTCCACAC	TGCTTGGGTAGTACAGGATGG	75
EGR1	CAATCCTTTCTGCCCACTTC	AGGTGAAGAACTTGGACATGG	27

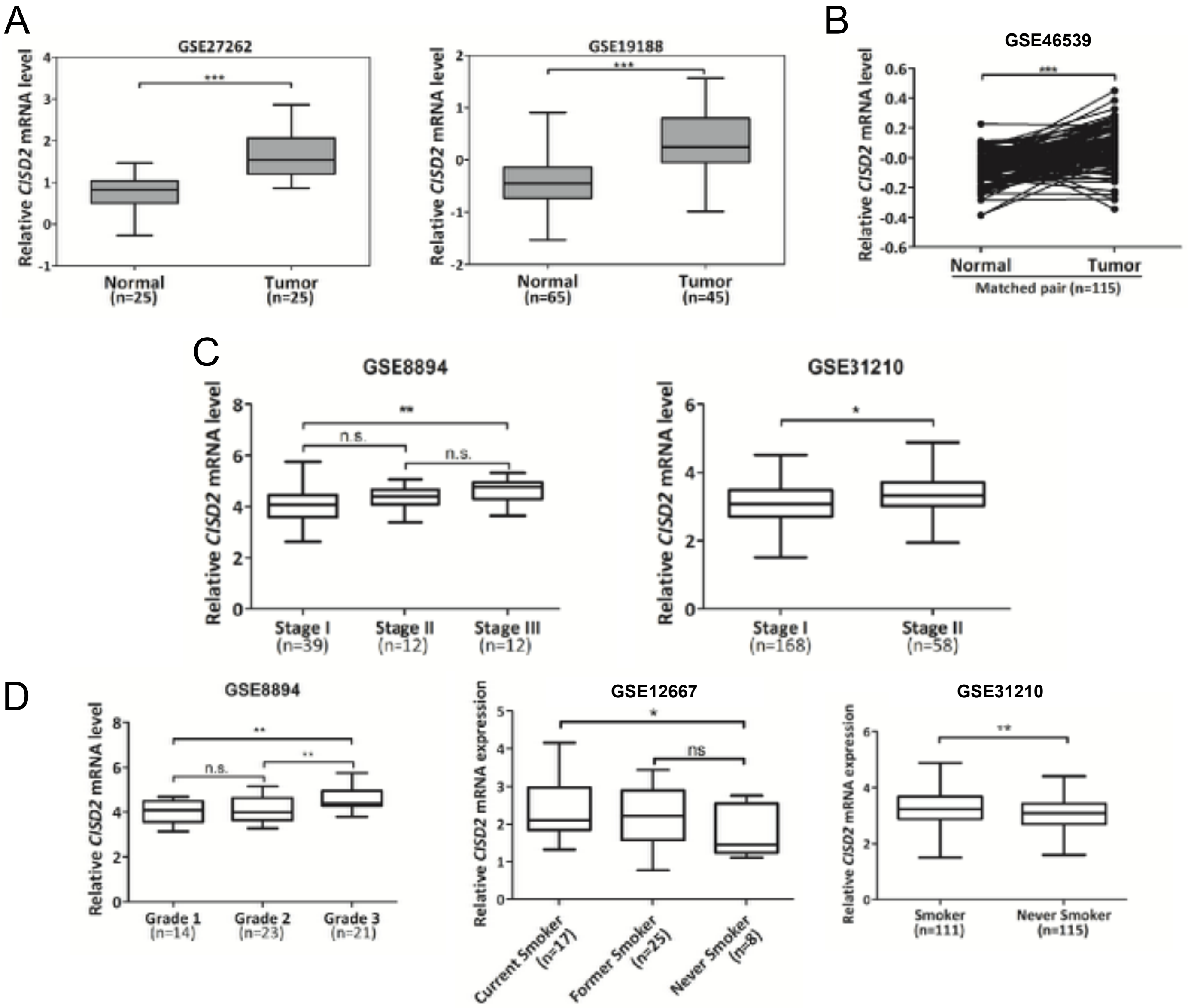

Supplementary Fig. S1. *CISD2* expression is upregulated in lung ADC and associated with patient status. (A) Box plot showing upregulation of *CISD2* mRNA expression levels in lung ADC tumor tissues compared with those in normal lung tissues, based on public domain data GSE27262 and GSE19188. Data were downloaded from Oncomine and analyzed directly without further processing. (B) *CISD2* mRNA expression levels in 115 matched normal-tumor pairs of lung ADC samples from our in-house generated dataset GSE46539. P values were obtained with paired t-test, ***P<0.001.(C) Box plots showing *CISD2* mRNA expression level is correlated with staging of lung ADC patients.(D) Box plots showing association between *CISD2* expression levels and grade or status of cigarette smoking. For (B), (C) and (D) P values were obtained using Student's *t*-test, *P<0.05, **P<0.01 and ***P<0.001. Detailed information about public domain datasets is available in Supplementary Table S1.

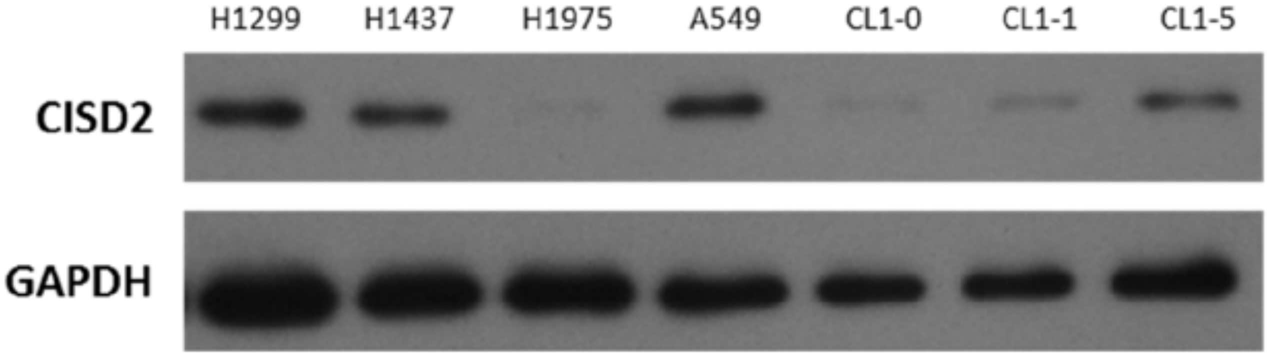

Supplementary Fig. S2. Western blot of CISD2 protein expression in several lung ADC cell lines used in this study.

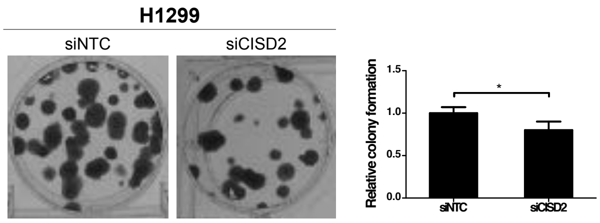

Supplementary Fig. S3. Colony formation assay of H1299 cells treated with siRNA of *CISD2*. *P<0.05.

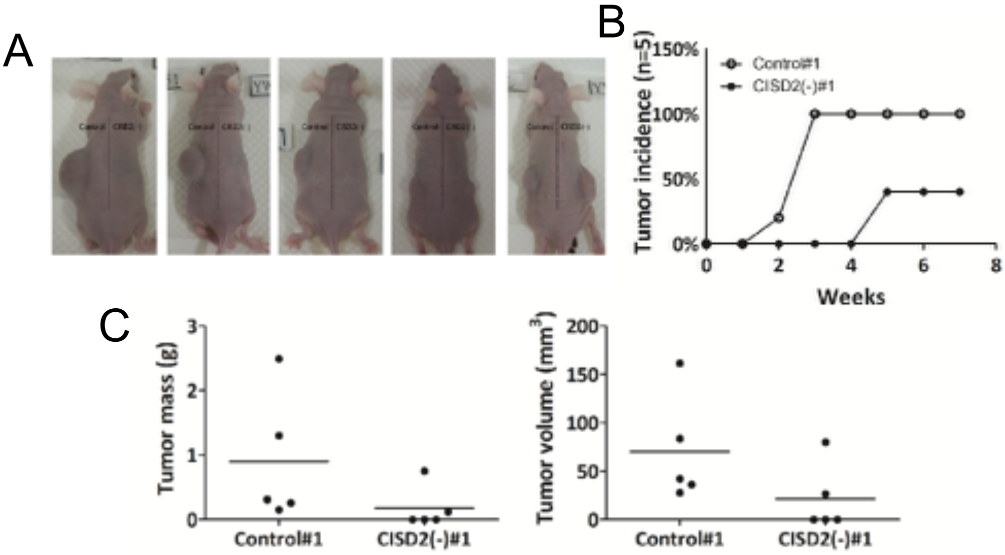

Supplementary Fig. S4. *CISD2* expression affects tumorigenicity of lung ADC cells *in vivo*. (A) Representative images of xenograft tumor growth on mice (n=5). (B) Kinetics of incidence of xenograft tumor formation during the period of experiment. The tumor incidence is defined as the percentage of mice (n=5) that have detectable tumor at a given time point. (C) Tumor mass (left panel) and tumor volume (right panel) of xenograft tumors formed by subcutaneous injection of CISD2(-)#1-H1299 cells into SCID mice (n=5 for each group) at the end point of the assay (seven weeks post injection).

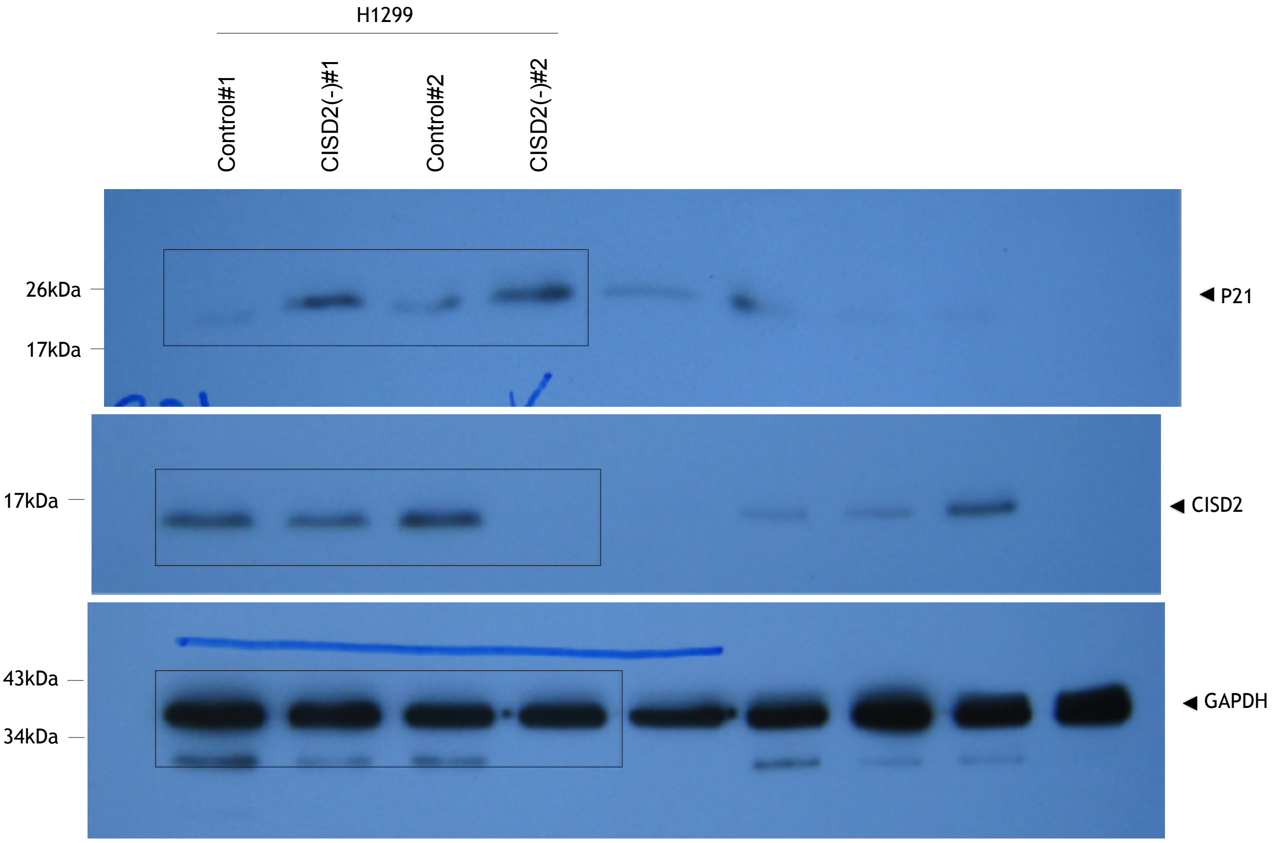

Fig. S5. Full images of unedited blots of Fig. 2C.

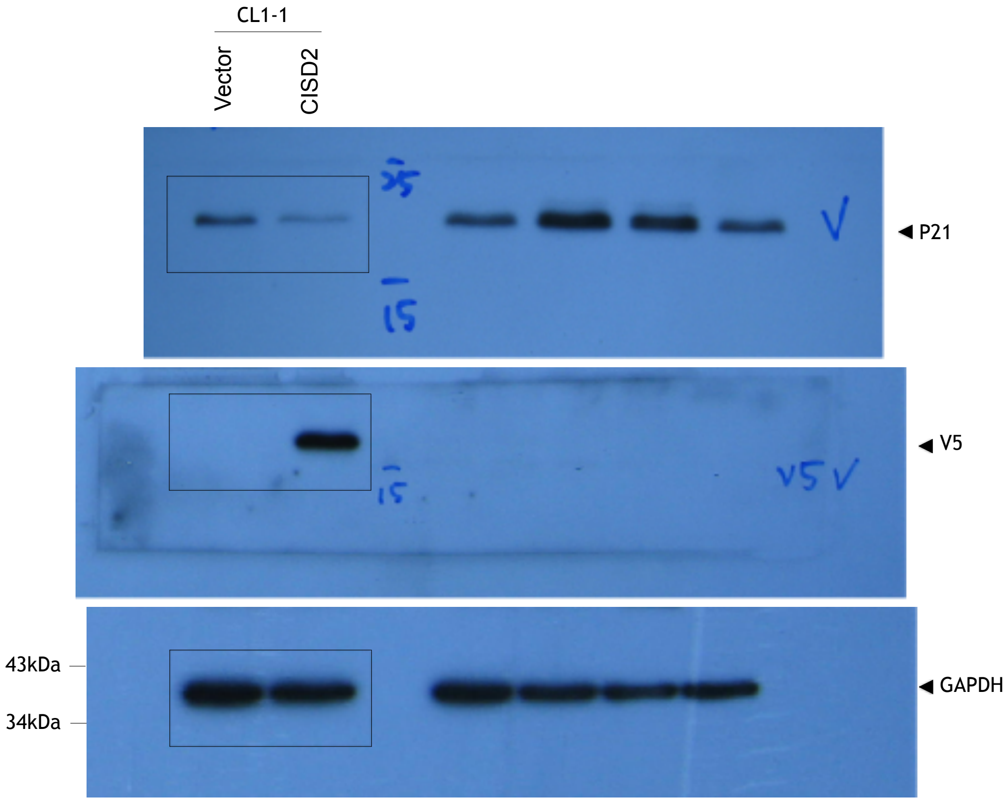

Fig. S6. Full images of unedited blots of Fig. 2D.

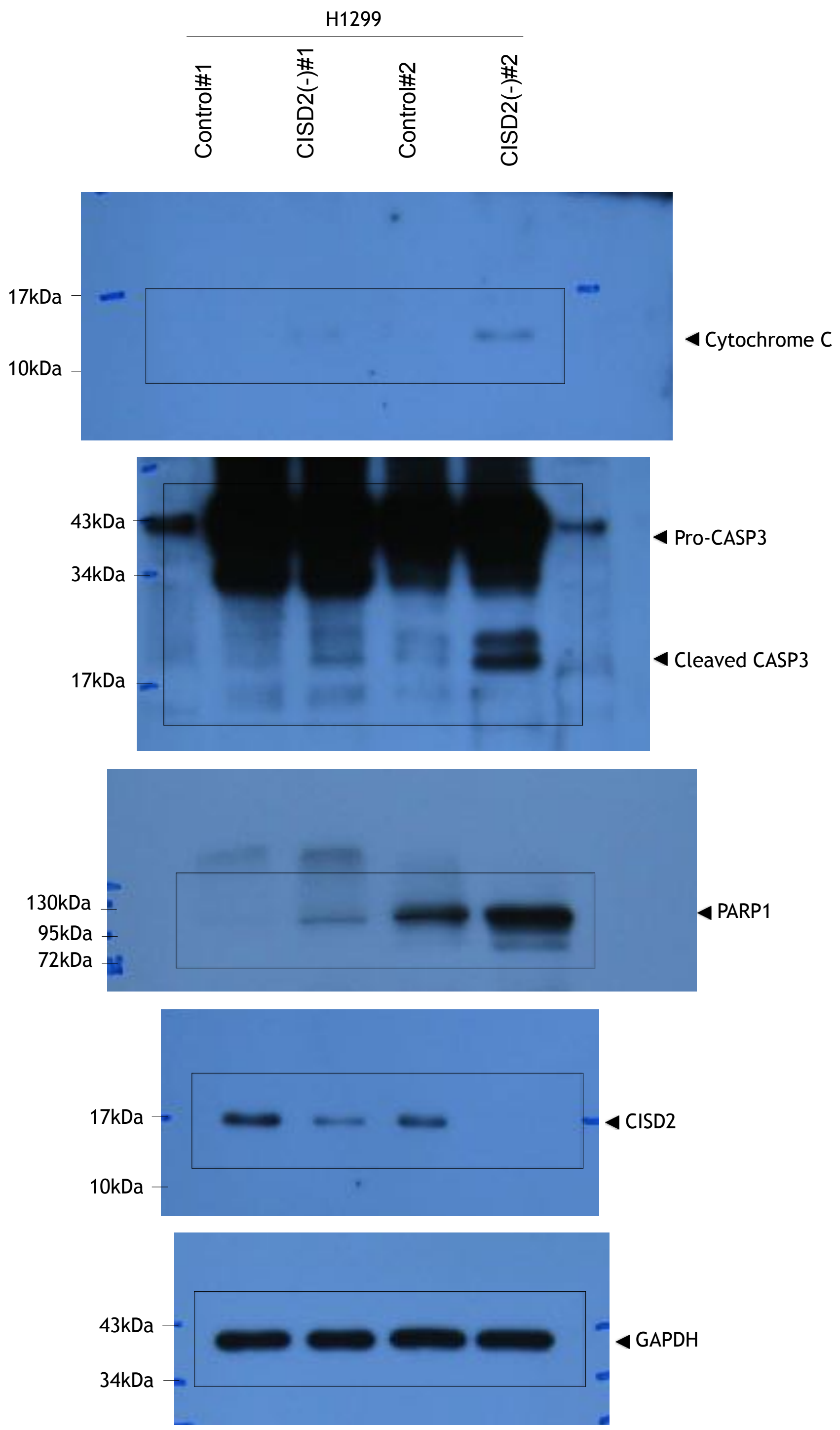

Fig. S7. Full images of unedited blots of Fig. 3B.

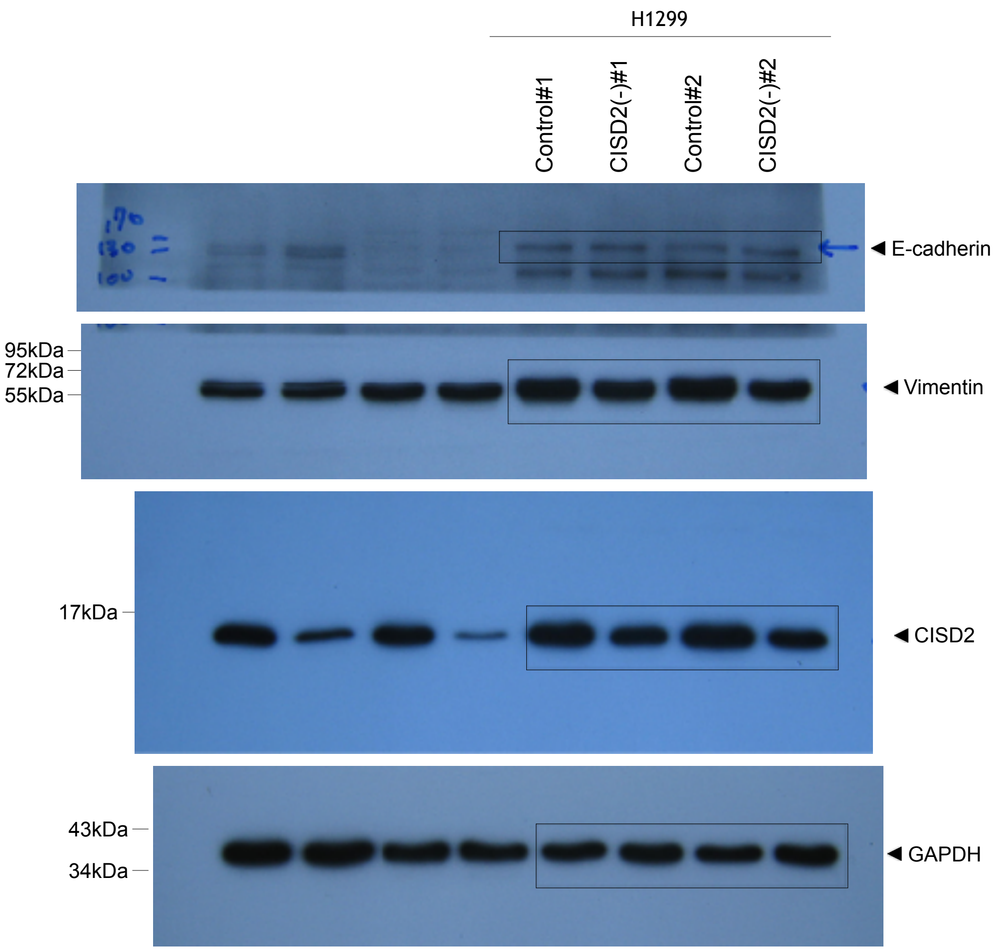

Fig. S8. Full images of unedited blots of Fig. 4D.

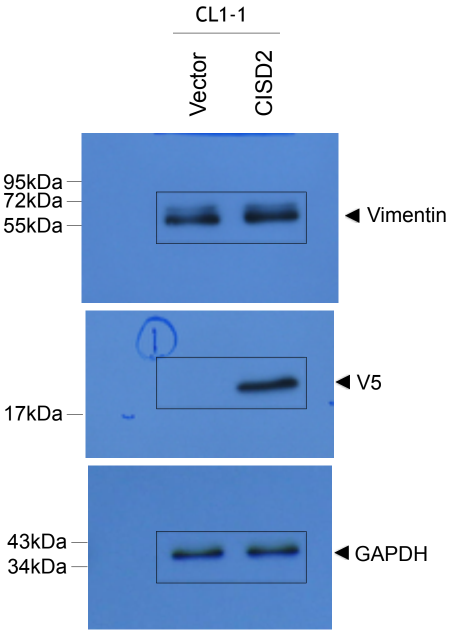

Fig. S9. Full images of unedited blots of Fig. 4F.

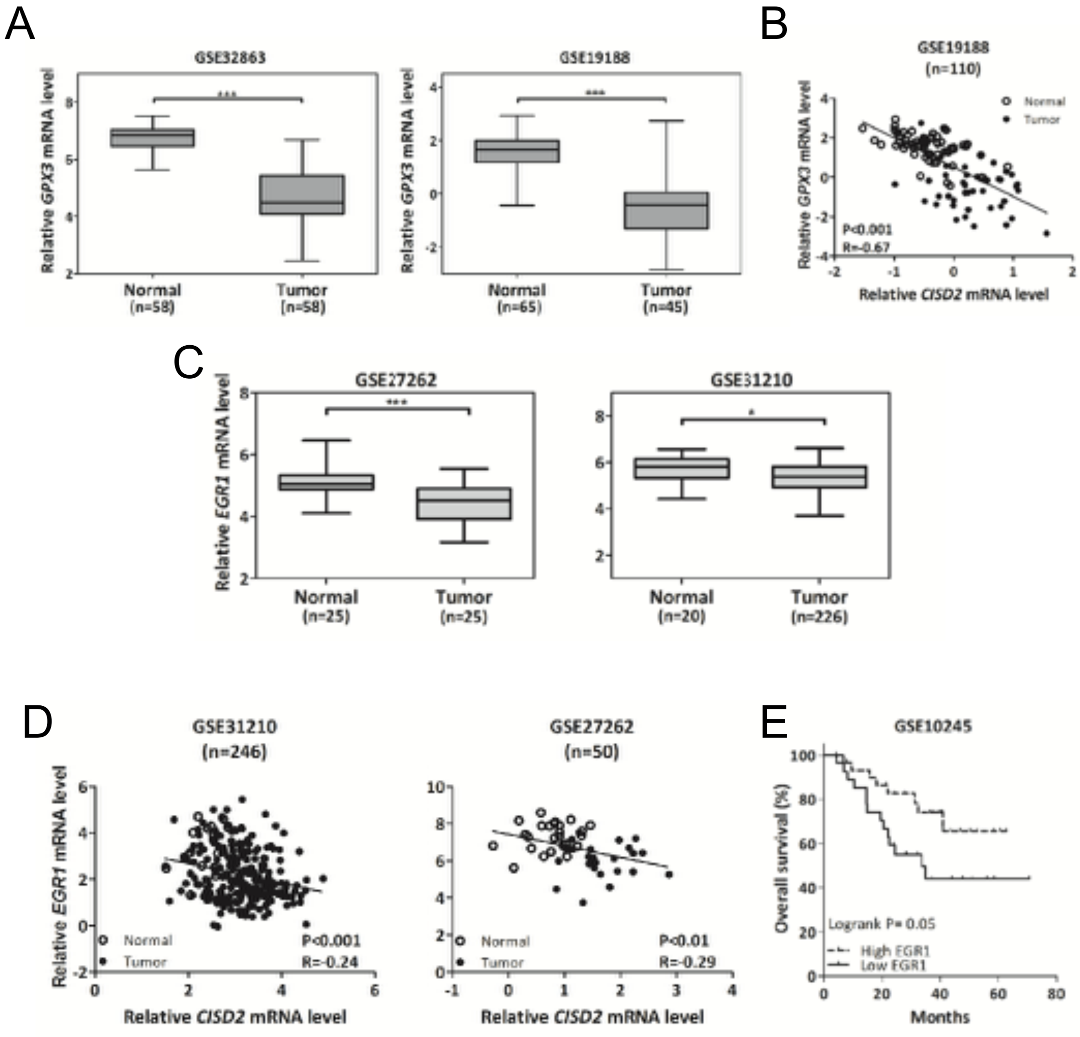

Supplementary Fig. S10. CISD2 negatively regulates GPX3 and EGR1. (A) Box plots showing *GPX3* mRNA expression profiles of lung ADC versus normal lung tissues extracted from public domain datasets. (B) Scatter plot showing significantly negative correlation between mRNA expression levels of *CISD2* and *GPX3* in GSE19188. Correlation test was used to estimate the significance of correlation. (C) Box plots showing *EGR1* mRNA expression profiles of lung ADC versus normal lung tissues extracted from two public domain datasets. (D) Scatter plots showing significant negative correlation between mRNA expression levels of *CISD2* and *EGR1* in two public datasets. (E) Survival analysis on *EGR1* mRNA expression and overall survival using public domain data. In GSE10245, patients were stratified into low *EGR1* (solid line) and high *EGR1* (dashed line) groups using median *EGR1* expression level as cutoff; significance of difference in survival between groups was estimated using log rank test. For (A) and (C) P values were obtained using Student's *t*-test, *P<0.05 and ***P<0.001. Detailed information about public domain datasets is available in Supplementary Table S1.

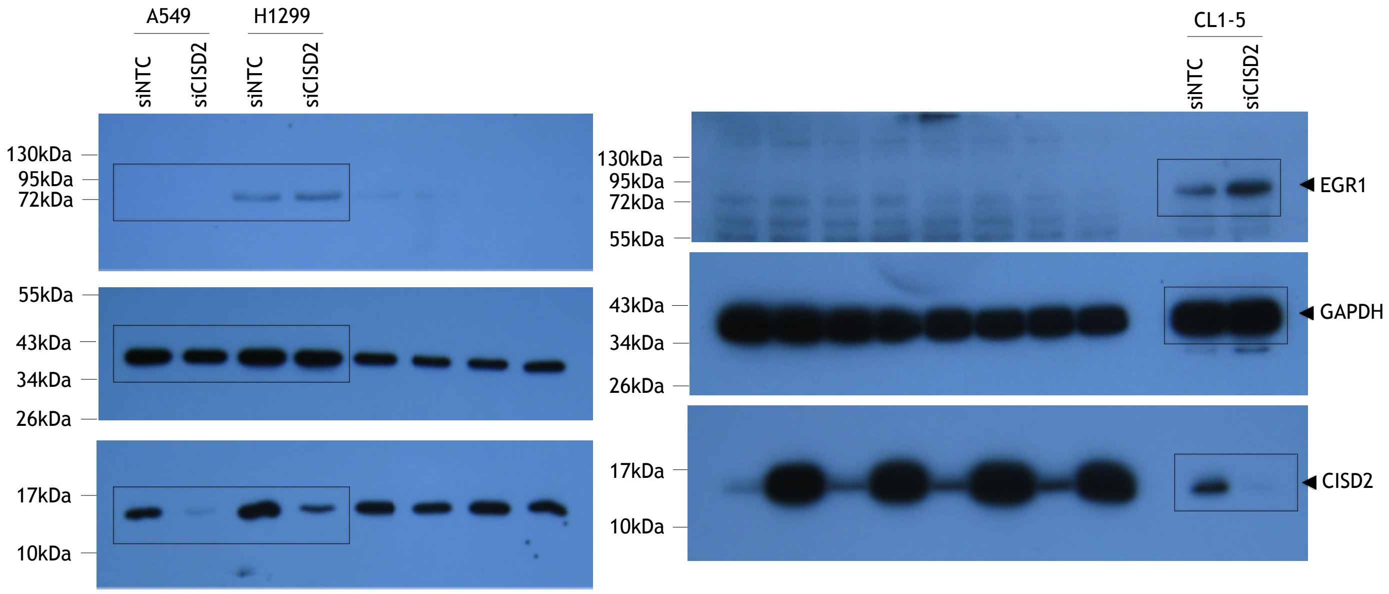

Fig. S11. Full images of unedited blots of Fig. 6J.

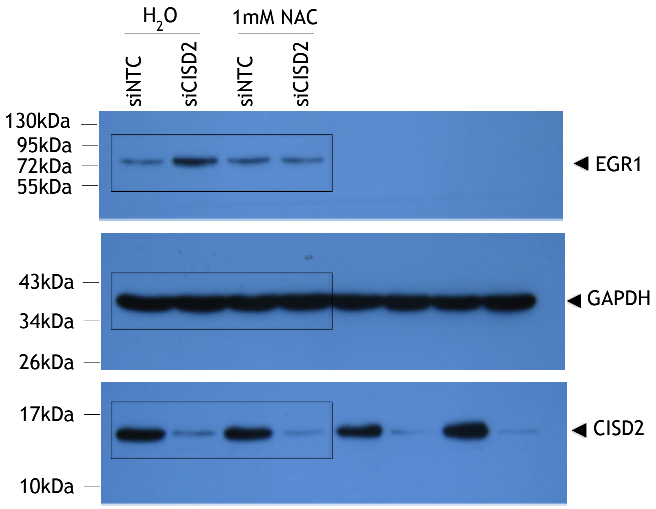

Fig. S12. Full images of unedited blots of Fig. 6K.

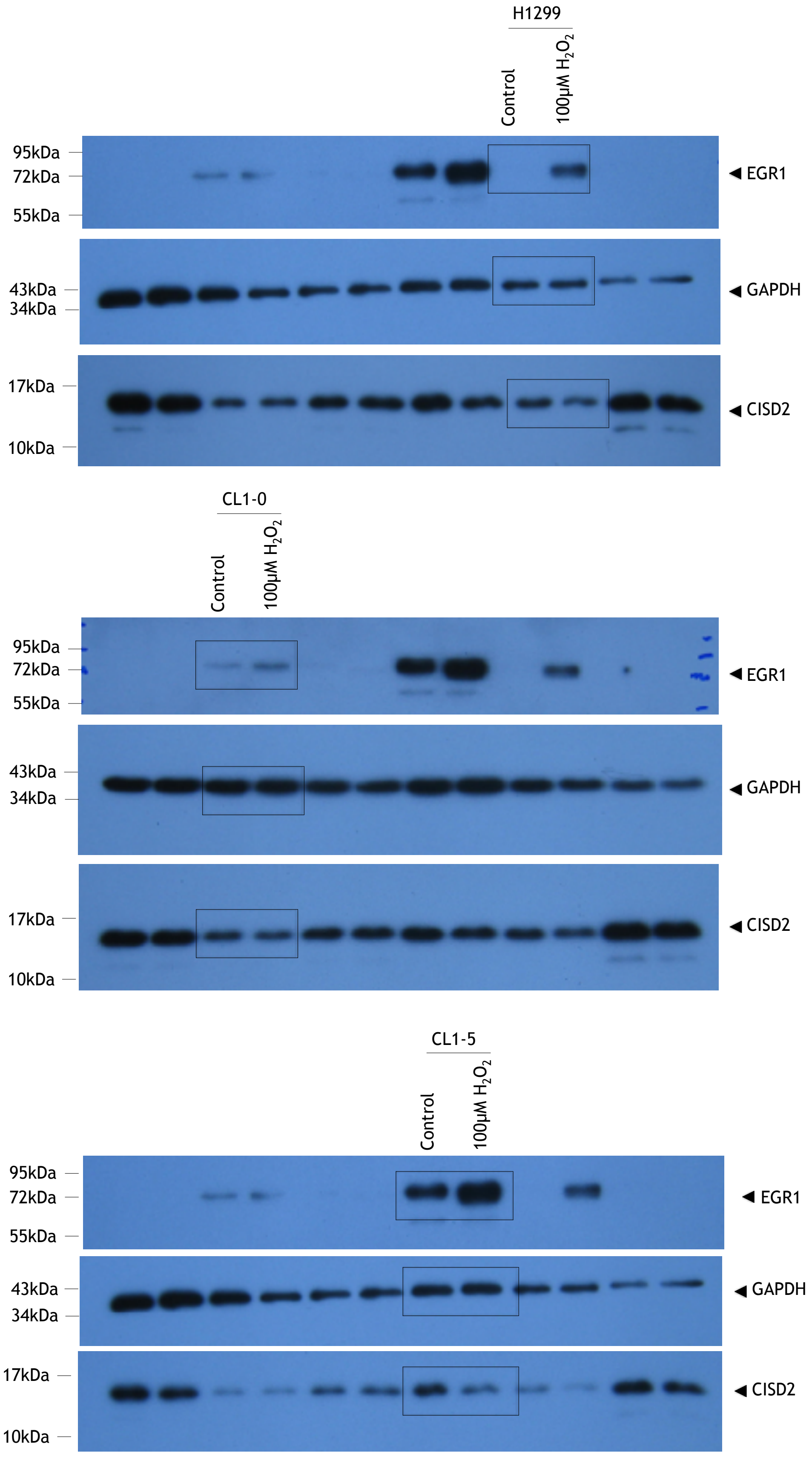

Fig. S13. Full images of unedited blots of 6L.

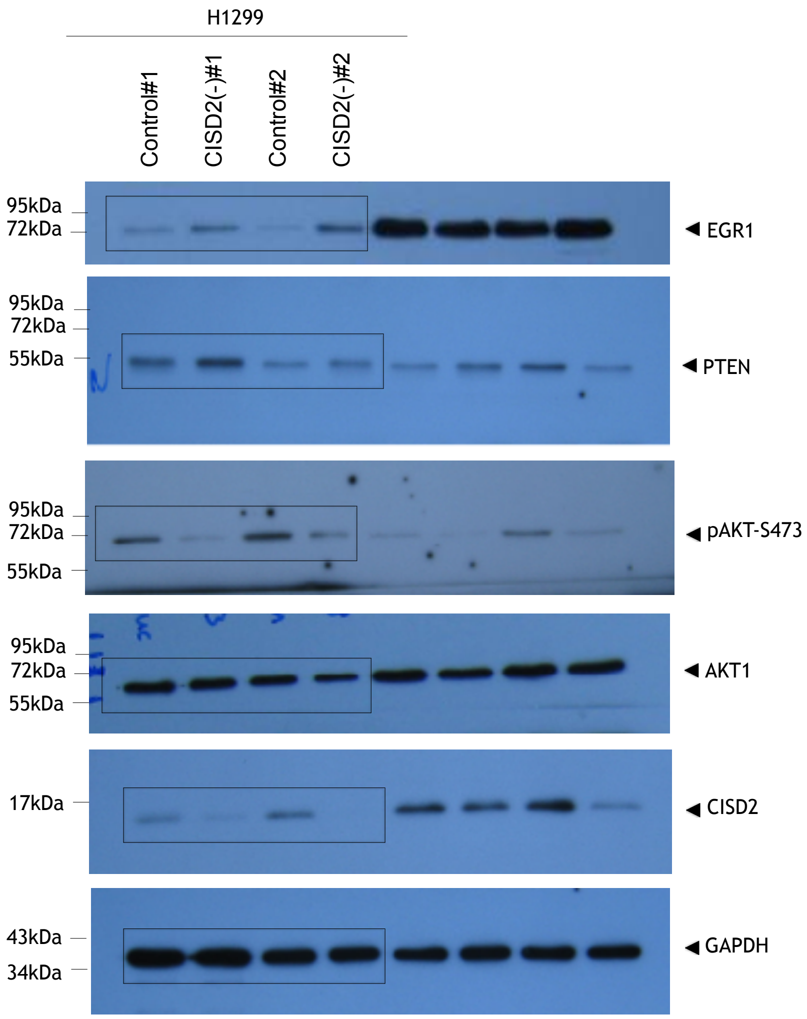

Fig. S14. Full images of unedited blots of Fig. 6N.
